# Flower color variation in *Digitalis purpurea*: Pollination and soil influences across native and introduced populations

**DOI:** 10.1002/ajb2.70186

**Published:** 2026-04-03

**Authors:** Sissi Lozada‐Gobilard, Pamela Espinoza Peñaloza, Giovanni Pascucci, Zainab Aliwi, Emilia Larsson, Mikkel Brydegaard, Øystein H. Opedal

**Affiliations:** ^1^ Department of Biology Lund University Lund 22362 SE Sweden; ^2^ Instituto de Ecología Universidad Mayor de San Andrés La Paz Bolivia; ^3^ Department of Life, Health and Environmental Sciences University of L'Aquila Via Vetoio ‐ Coppito L'Aquila 67100 Italy; ^4^ Department of Physics Lund University Lund 22362 SE Sweden

**Keywords:** Andes, Bolivia, Bombus, flower color, foxglove, pollination, soil characteristics, Sweden

## Abstract

**Premise:**

Flower color, a key trait influencing plant–pollinator interactions, may be influenced by abiotic factors such as soil. We investigated association between pollinators, soil characteristics, and flower color variations in *Digitalis purpurea* across native populations in Sweden and introduced populations in Bolivia.

**Methods:**

We measured floral traits, reflectance of petals and nectar‐guide spots, plant size, pollinator visitation, fruit set, seed production, germination, and soil characteristics.

**Results:**

Individuals were categorized into violet, pink, and white morphs, which were confirmed by spectral measurements and bee vision modelling. Reflectance of inner nectar‐guide spots overlapped across morphs, potentially limiting pollinator discrimination. Bumblebees were the main pollinators in all populations. Although visitation varied among morphs, pollinator visits to different color morphs were population specific. In Bolivia, violet flowers were predominant (70–87%), with pink (13–17%) and white (0–13%) at lower frequencies. In Sweden, morph frequencies were more even (violet 20–43%, pink 38–69%, white 11–30%). Morph frequency was not associated with soil composition, despite differences between regions. Reproductive fitness varied across populations but not consistently among morphs: The largest Swedish population had the highest fruit set but the lowest seed set, while germination was lower in Bolivia. Phosphorus was lower in soil beneath violet individuals; other soil variables, plant size, and floral traits did not differ among color morphs.

**Conclusions:**

Floral color variation in *D. purpurea* was not significantly related to pollinator visitation or soil conditions at the spatial scale examined, suggesting maintenance by local environmental conditions, neutrality, or historical and demographic processes rather than selection.

Flower color varies among species, populations, and individuals, yet the causes of this variation are not always clear and may include both biotic and abiotic factors (Sapir et al., 2021). Color is an essential visual advertisement toward pollinators (Narbona et al., 2021; van der Kooi et al., 2021), and their preferences for certain colors indirectly affect plant fitness (Jones and Reithel, 2001; Dormont et al., 2010), hence influencing pollinator‐mediated selection on flower color (reviewed by Trunschke et al., 2021). At the same time, colorful flowers can attract antagonists feeding on leaves and flowers (Vaidya et al., 2018; Saabna et al., 2024). Furthermore, abiotic conditions such as temperature (Nozaki et al., 2006), soil nutrients, pH (Kodama et al., 2016), water availability (Vaidya et al., 2018), and tissue structure (van der Kooi et al., 2014; Stavenga et al., 2020) can also affect flower color at the individual and population levels.

While pollinators are often considered the primary drivers of floral trait evolution, a growing body of evidence highlights the importance of nonpollinator selection agents in shaping and maintaining floral color polymorphism (Strauss and Whittall, 2006). Abiotic factors such as temperature, UV radiation, soil nutrient availability, and water stress can exert strong selective pressures on flower color by influencing pigment production and stability. In particular, flower colors associated with higher anthocyanin concentrations can improve plant tolerance to heat, drought, and intense UV radiation, thereby enhancing fitness under stressful environmental conditions (Warren and Mackenzie, 2001; Winkel‐Shirley, 2001; Arista et al., 2013; Ortiz et al., 2015; Berardi et al., 2016; Sullivan and Koski, 2021). These physiological advantages can drive geographic variation in flower color, leading to the segregation of color morphs across environmental gradients and contributing to the maintenance of color polymorphism within species (Strauss and Whittall, 2006; Sapir et al., 2021).

Soil pH and nutrient composition can determine flower color by influencing anthocyanin synthesis and pigment stability. Key elements such as nitrogen, phosphorus, potassium, aluminum, and iron play crucial roles in modifying pigmentation. For example, acidic soils with high aluminum (Al) content are associated with blue hues, while alkaline conditions favor pink or red shades (e.g., Toyama‐Kato et al., 2003; You and Yoo, 2023). An increase in the concentration of iron in the soil can induce a color change to blue (Li et al., 2024), while soil deficiencies in potassium and phosphorus can lead to an increase in flower pigment concentration (Chen et al., 2013; Vaidya et al., 2018). Soil conditions, including pH and ion availability, can also influence flower color through phenotypic plasticity, as in the case of *Hydrangea*, in which aluminum availability in acidic soils induces blue flowers, while alkaline soils lead to pink flowers (Schreiber et al., 2011; Yoshida et al., 2020). At the same time, soil conditions may act as selective agents on genetically based petal color variation if certain colors confer advantages in a specific abiotic environment (Warren and Mackenzie, 2001; Strauss and Whittall, 2006; Labin et al., 2026). This distinction highlights the dual role of soil factors in shaping floral color—via phenotypic plasticity and via selection on heritable traits.

Foxglove, *Digitalis purpurea*, is a biennial herb native to western Europe that has been introduced to America, Asia, and Australia likely through multiple independent events (Bräuchler et al., 2004; Mackin et al., 2021b). The bell‐shaped flowers are pollinated mainly by *Bombus hortorum* in its native range (Grindeland et al., 2005; Broadbent and Bourke, 2012). In native populations in the Netherlands, flowers are usually violet, with a very low proportion (0.5%) of white‐flowered individuals, and pollinators do not exhibit a strong preference for any specific flower color morph (Ernst, 1987). However, white‐flowered individuals have produced fewer seeds and germination rates are lower than for violet‐flowered plants (Ernst, 1987). Additionally, the white‐flowered morph has a higher demand for water and soil nutrients, especially phosphorus (Ernst, 1987).

The intense violet color of *D. purpurea* flowers arises, like in most violet/red‐flowered species, from anthocyanins (Wolff et al., 2024 [preprint]). The complete loss of color in white flowers is likely due to a disruption in anthocyanin biosynthesis, specifically affecting the anthocyanidin synthase (ANS) enzyme for which white plants contain a homozygous insertion of a DNA fragment (~13 kb), while the violet flowers have at least one allele without this insertion (Wolff et al., 2024 [preprint]). However, in cultivation and in nature, flowers show a wide range of hues—from white and pink to bright violet—suggesting a role of anthocyanin concentration in determining flower color intensity.

Flowers of *Digitalis purpurea* also vary in color in both native and introduced populations. The principal pollinators in the native range are bumblebees, while a shift in the shape of the corolla base in introduced populations in South America is associated with adaptation to hummingbird pollination, although insect pollination also occurs (Mackin et al., 2021b). Comparing native‐range to introduced populations provides a valuable opportunity to investigate whether flower color variation is shaped by similar or divergent biotic and abiotic drivers across contrasting environments. Differences in pollinator assemblages, local herbivore pressures, and climatic and edaphic conditions can contribute to changes in selective regimes and potentially influence the maintenance or evolution of color polymorphism.

Here, we investigated how biotic (pollinator visitation) and abiotic factors (soil characteristics) influence flower color variation in *D. purpurea* at the individual level within and across native‐range (Sweden) and recently introduced (Bolivia) populations. We hypothesize that, in native populations, flowers will be primarily pollinated by *Bombus* species, with no strong preference for a particular flower color morph as previously found by Ernst (1987). This lack of preference may be partly explained by the presence of dark spots on the corolla and trichome‐like structures, which act as nectar guides to direct pollinators toward the reward (Lunau, 1993; Whitney and Glover, 2007; Hansen et al., 2012; Doody and Moyroud, 2025). Indeed, some studies have shown that nectar guides can be more important than petal color in directing pollinator behavior (Penny, 1983; Waser and Price, 1985; Hempel de Ibarra et al., 2015). We also predict that soil nutrient availability—particularly phosphorus, nitrogen, potassium, and iron—will influence plant vigor and potentially affect floral color expression, as nutrient deficiencies can constrain anthocyanin production (Winkel‐Shirley, 2001; Chen et al., 2013; Vaidya et al., 2018). Because Ernst (1987) found higher phosphorus uptake in white‐ compared to violet‐flowered plants, we expect that variation in soil parameters may be associated with differences among color morphs, and consequently, with reproductive success (Rodríguez‐Castañeda et al., 2020; Buide et al., 2021; Sapir et al., 2021).

## MATERIALS AND METHODS

### Study species

*Digitalis purpurea* L. (Plantaginaceae) is a facultative biennial, semelparous herb that grows in forest gaps and other disturbed sites. Upon flowering, each individual produces one or a few inflorescences (1–2 m tall), with 20–100 flowers. The flowers are self‐compatible, bell‐shaped, and protandrous, with stamens releasing pollen shortly after anthesis. Approximately 5 days later, the stigma becomes receptive (Darwin, 1876). Although plants are self‐compatible, full seed set requires insect visitation (Nazir et al., 2008; Mackin et al., 2021b). Flowering proceeds acropetally, with basal flowers maturing first. Nectar production increases over the flower's lifespan (Percival and Morgan, 1965), encouraging bumblebees to initiate foraging at the lower, older flowers.

*Digitalis purpurea* has become naturalized across many temperate regions and tropical highlands worldwide (Bräuchler et al., 2004). In South and Central America, populations likely originated from garden escapees introduced by British settlers around the 1850s (Calle et al., 1989; Mackin et al., 2021b). In Bolivia, it was probably introduced during the same period and has been documented in traditional medicinal use by the Kallawaya, an Indigenous Amazonian group renowned for their pharmacological knowledge (Janni and Bastien, 2004). In its native range, including southern Sweden, *D. purpurea* blooms during summer, from late June to August, and is typically biennial flowering after 2 years. In introduced regions, it flowers mainly from September to February, peaking during the wet season between December and January. However, it is unclear whether the species remains biennial in the introduced range.

Flowers have distinct spots of dark violet bordered by white on the lower petal, which act as nectar guides (Lunau, 1993). The deep violet spots are perceived as purple by humans and green by bees, due to their absorption of ultraviolet and green wavelengths and reflection of blue and red light (Lunau et al., 2024). Additionally, it has been proposed that the violet spots in *D. purpurea* act as anther mimics, potentially guiding pollinators toward the reproductive organs (Lunau et al., 2024).

### Study sites

Here we studied five populations of *D. purpurea*, three in southern Sweden (within the native range), and two in the tropical Andes of Bolivia where it is introduced. The native populations were located in Scania County in southern Sweden, two near Höör (H1, H3) and one near Genarp (G1; Appendices S1, S2). This region has an oceanic climate with mild winters (–1° to 5°C) and cool to warm summers (15°–25°C). The annual precipitation (600–1000 mm) is evenly distributed year‐round, with wetter months in late autumn. Winters are cloudy with short days, while summers have long daylight hours. Coastal influence keeps temperatures moderate compared to the rest of Sweden.

The introduced populations are located in the Yungas region of Bolivia (Appendices S1, S2). The Yungas region, which mainly belongs to Cotapata Parque Nacional‐Area Natural de Manejo Integrado (PN‐ANMI), is a hotspot of biodiversity with an altitudinal variation from ~1500 m to 3000 m a.s.l. This region is characterized by three vegetation belts: submontane forest (<1500 m a.s.l.), humid montane forest (1500–2400 m) and cloudy forest (2400–3500 m). We studied two roadside populations in the cloud‐forest zone. This type of forest has a warm and humid climate with high precipitation. Temperatures average around 22°C but drop to freezing at higher altitudes above 2000 m. Annual rainfall varies from 2300 to 5300 mm, with highest values during the rainy season from February to April. The terrain is steep and rugged, contributing to a rich biodiversity including epiphytes like bromeliads and orchids.

### Flower color and morphology

**Figure 1:**
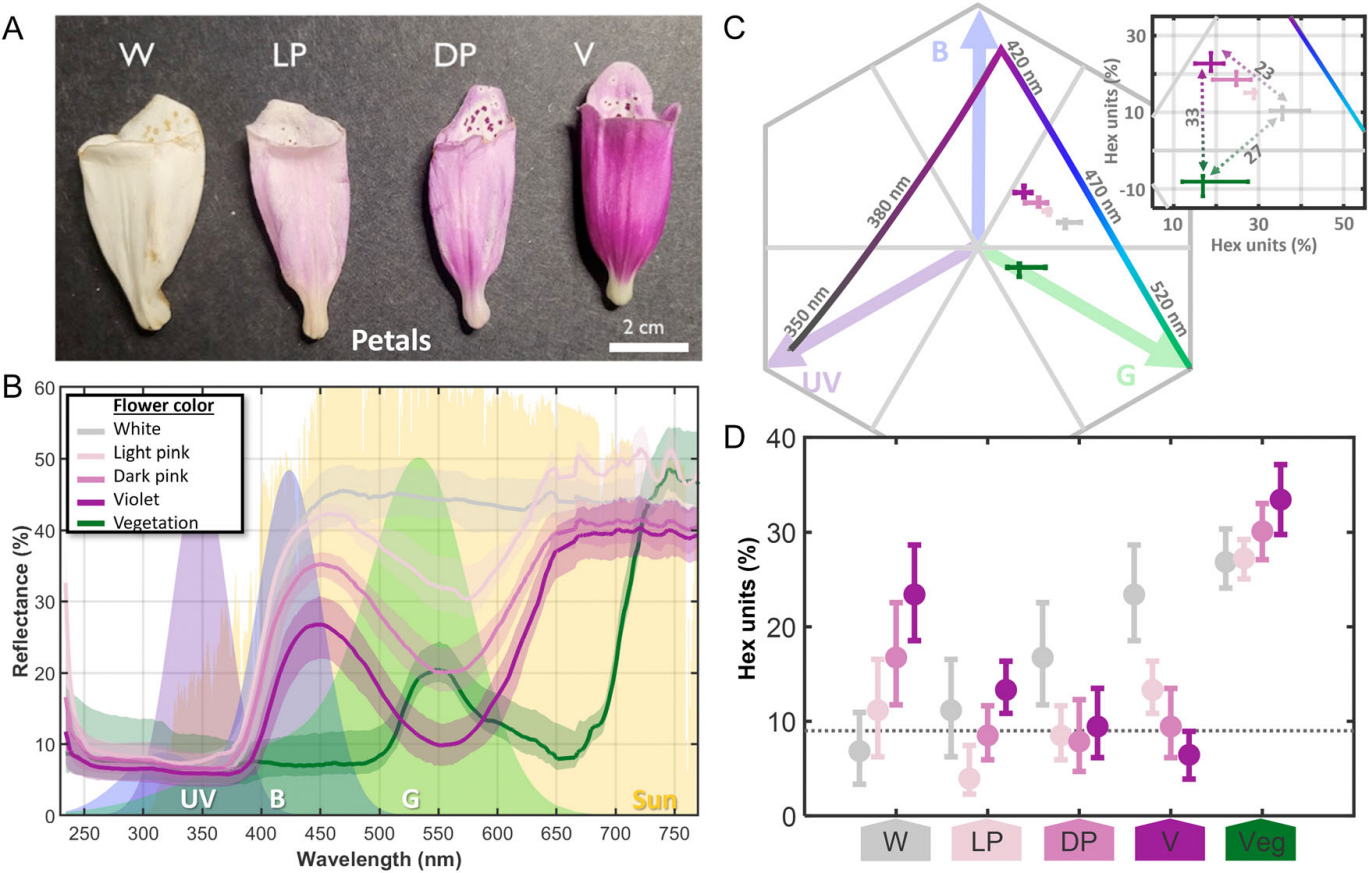
Floral reflectance and bee vision model from petals of *Digitalis purpurea*. (A) Flowers were categorized into four groups: white (W), light pink (LP), dark pink (DP), and violet (V). (B) Petal reflectance spectra for each color category (*N* = 5 violet, 7 dark pink, 3 light pink, 5 white). Reflectance of green vegetation is shown in dark green. The solar irradiance and spectral receptor bands (λ peak: 347, 424, and 539 nm) are shown; values are in arbitrary normalized units of *Bombus terrestris* (Skorupski and Chittka, 2010). (C) Bee hexagon for *D. purpurea* flowers; center represents the achromatic center, and edges show maximum excitation for blue, green, and UV photoreceptors. Colored dots represent the average of each flower color category. (D) Median chromatic distance in hexagon units between each floral color category pairwise comparison. The vertical bars indicate the interquartile range (IQR) among all combinations. The dashed line represents the bee discrimination threshold (9% hexagon units); points below the line are likely to be perceived equally in bee vision.

We initially assigned individuals to one of four color groups: violet (V), dark pink (DP), light pink (LP), and white (W) (Figure 1A). Color groups were first classified by eye in the field and later confirmed by reflectance measurements. Although four visually distinct morphs were identified (Figure 1B), the reflectance spectra of light and dark pink individuals were very close in color space (Figure 1C), indicating that these categories represent variants within a continuous pink range rather than distinct clusters. In addition, the separation between LP and DP was often ambiguous and inconsistent under field conditions, especially during pollinator observations. For these reasons, and to ensure sufficient sample sizes and robust statistical analyses, we combined light and dark pink into a single “pink” category for all downstream analyses.

**Figure 2:**
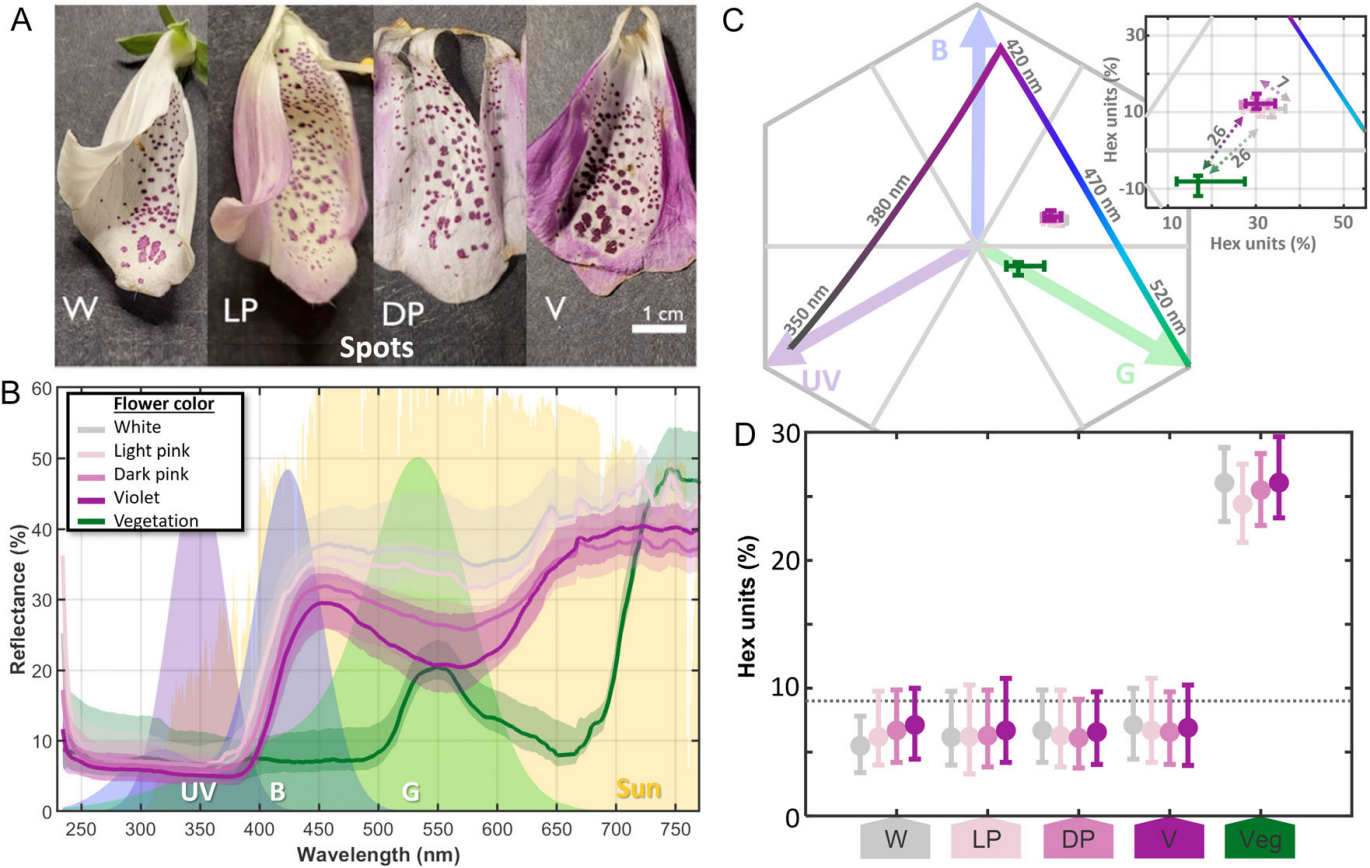
Floral reflectance and bee vision model from inner spots of *Digitalis purpurea*. (A) Reflectance from inner spots of white (W), light pink (LP), dark pink (DP), and violet (V). (B) Inner spots (i.e., nectary guides) reflectance spectra for each color category (*N* = 5 violet, 7 dark pink, 3 light pink, 5 white). Reflectance of green vegetation is shown in dark green. The solar irradiance and spectral receptor bands (λ peak: 347, 424, 539 nm) are shown; values are in arbitrary normalized units of *Bombus terrestris* (Skorupski and Chittka, 2010). (C) Bee hexagon for *D. purpurea* inner spots. Center represents the achromatic center; edges show maximum excitation for blue, green, and UV photoreceptors. Colored dots represent the average of each flower color category. (D) Median chromatic distance in hexagon units between each floral color category pairwise comparison. Vertical bars indicate the interquartile range (IQR) among all combinations. Dashed line represents the bee discrimination threshold (9% hexagon units), where points below the line are likely to be perceived equally in bee vision.

Nectary‐guide spots located in the lower lip of the flowers are mostly dark violet surrounded by a white shade and are present in all color morphs (Figure 2A), and their reflectance spectra overlapped considerably, particularly between dark pink and violet flowers (Figure 2B). However, we also observed a few white‐flowered individuals, with spots that were faintly pigmented or entirely absent.

To characterize flower morphology, we randomly selected three flowers from different parts of the inflorescence and measured their morphology in the field using digital calipers. We measured flower length, flower width, proximal corolla width and length. We calculated the area of the whole corolla and proximal part of the corolla by multiplying width and length of the flower and the proximal corolla, respectively, using the methodology of Mackin et al., (2021b). Additionally, we recorded whether the flower was robbed, as indicated by small wounds at the side of the proximal corolla.

### Floral reflectance and bee vision

We measured spectral reflectance on a subset of plants of all color morphs from fresh flowers (3–5 days of anthesis corresponding to the male phase) from the Swedish populations (*N* = 20: G1, 10; H1, 7; H3, 3), confirming the visually assigned color categories (Figure 1A, B). The measurements were made with a bifurcated fiber that collected reflectance at normal incidence from a working distance of 5 mm and a probe spot diameter of ~2 mm. The light delivery fiber was coupled to a deuterium tungsten broadband UV light source (Thorlabs, Newton, NJ, USA). The collection fiber was coupled to a spectrometer with a useful spectral range of 230–830 nm and a resolution of 10 nm FWHM (full width at half maximum). The spectrometer includes a filter rejecting higher‐orders diffractions. Spectral calibration was done with a Cd lamp, and flat field calibration was done with a diffuse grey reflectance standard (LabSphere, North Sutton, NH, USA).

We took three random flowers per plant and recorded three reflectance measurements per flower, selecting both petal surfaces and nectar guides. For petal color, measurements were taken from randomly chosen smooth and uniformly colored areas of the corolla to ensure consistent spectral readings. For the inner nectar guides, we selected the largest and most clearly defined pigmented spots. However, because the size of most of the spots were smaller than the spectrometer probe (2 mm spot size), the resulting measurements spatially averaged reflectance of the target spot with that of the adjacent white petal tissue. Reflectance measurements were performed at the Department of Physics at Lund University.

To assess variation in pollinator‐perceived floral chromaticity, we projected the reflectance spectra of the flowers on the hexagonal visual sensitivity model developed for bees by Chittka (1992) using the bumblebee color bands of *Bombus terrestris* (Skorupski and Chittka, 2010). The contribution to the spectral receptors was calculated as:

$$E_{\text{band}}=\frac{\int R_{\text{flower}\left(\lambda \right)}S_{\text{band}\left(\lambda \right)}I_{\text{sun}\left(\lambda \right)}d\lambda }{\int S_{\text{band}\left(\lambda \right)}I_{\text{sun}\left(\lambda \right)}d\lambda },\;\text{band}\in \left\{\mathrm{UV},\mathrm{blue},\mathrm{green}\right\}$$

where *E*_band_ is the contribution of a flower reflectance to the UV, blue, or green band; *R* is the spectral reflectance recorded from the flowers; *S* is the sensitivity curve for the particular band (Skorupski and Chittka, 2010); and *I*_sun_ is the sunlight at ground level with the standard 1.5 atmosphere model. (The effect of including sunlight is the cut‐off below 310 nm where the ozone layer block natural sunlight.) The chromaticity was calculated as the relative contributions of the spectral vision bands and projected on Chittka's hexagonal ultraviolet–blue–green (UBG) color space (see Figure 1C). A standard adaptation background was implemented in the model to calculate the position of each color morph (Chittka and Menzel, 1992; Chittka et al., 1994). For convenience, we added the trajectory of monochromatic light (maximal possible chromatic saturation) to the same figure and also added background vegetation characterized by spectral recordings (*N* = 3) of 24 green leaves from grass (*Lolium perenne*), dandelion (*Taraxacum officinale*), clover (*Trifolium* sp.), goutweed (*Aegopodium podagraria*), and daisy (*Bellis perennis*).

Given that *Bombus* species are the principal pollinators of *Digitalis purpurea*, we used the visual system of *B. terrestris* as a model (Skorupski and Chittka, 2010) to evaluate whether observed differences in floral reflectance are likely to be perceived by bees. Bumblebees can discriminate colors when the chromatic distance between stimuli exceeds 0.09 (9%) hexagon units (Dyer, 2005) indicated by the dashed lines in Figures 1, 2. Chromatic contrast was quantified as the Euclidean distance from each stimulus to the achromatic center of the hexagon (Chittka, 1992).

### Plant size traits

In each population, we marked around 45 individual plants (Appendix S1). An individual plant can have one vegetative rosette and one to several (many) inflorescences or be defined as a patch where more than one individual grows closely together and are not easily distinguished. We measured the diameter of the rosette and total inflorescence height. If an individual had more than one inflorescence or appeared to be several individuals growing closely together, we considered it as a patch and calculated the average per individual/patch.

### Pollinator observations

To estimate and compare visitation rates among color morphs, we made repeated 5–10‐min pollinator censuses for a total of 2–6 h of observations per population. For each repetition, we observed 15–20 individual plants per population, and an average of 20 min per plant. We observed all pollinator visits to flowers and recorded the number of inflorescences and the corresponding color morph (white, pink, violet). When observing pollinators, we estimated the number of individuals based on three color categories: violet, pink or white. We then estimated the number of visits per minute and calculated an “adjusted” visitation rate to account for the different flower proportions observed within each population:

$${\text{Visitation}}_{\text{Adj}}=\frac{\text{Visitation rate}}{\text{Prop}},$$

where Visitation rate is the number of visits per minute observed on a particular color morph (violet, pink or white) and Prop is the proportion of observed individuals of a particular color morph (violet, pink, or white) in the population. In other words, these adjusted visitation rates represent the visits of the pollinators in an ideal scenario where all the color morphs are equally available.

During the 6 h of observations of the Bolivian B2 population, we failed to observe hummingbirds visiting *D. purpurea*. Occasional observations of hummingbird visits to violet flowers were recorded in the previous season of 2023–2024 at the same population (15 visits in 2 h). We observed some hummingbirds flying nearby and visiting *Centropogon* sp. flowers, but they did not visit *Digitalis* flowers. Therefore, we calculated pollinator visitation rate adjusted by flower color based only on visits of long‐tongued bumblebees (*Bombus*), the most‐abundant pollinator group in all populations.

### Reproductive fitness

We revisited all marked individual plants after flowering and counted all fruits on each inflorescence and collected 3–5 fruits from different heights. Fruits were dried at air temperature, and later seeds were counted using ImageJ (Schneider et al., 2012). We calculated the number of fruits per individual by multiplying the number of fruits per inflorescence by the number of inflorescences per individual plant. Seed set was defined as the number of seeds per fruit per individual plant. Unfortunately, due to restricted accessibility caused by heavy rain at the end of the season, we could collect fruits only from one (B1) of the two introduced populations in Bolivia.

### Germination assay

We randomly selected 1–3 fruits from each of the surveyed individual plants from the populations B1, H1, H3, and G1. From each fruit, we sowed 30 seeds in 0.5‐L pots filled with peat‐free soil and topped with a thin layer of vermiculite. Approximately 10 mL of fungicide was applied to each pot, which was then placed in plastic trays. The trays were watered every 2–3 days and kept under LED light the first 2 weeks (Appendix S3). Once germination began, we counted the number of seedlings every 2–3 days over 25 days to assess seed viability. At the end of the assay, we recorded the total number of germinated seeds and calculated germination rate as the proportion of germinated seeds, averaged per fruit and individual.

### Soil sampling and analysis

Soil samples were collected from three Swedish populations (native) and two Bolivian populations (introduced). Soil sampling followed an adapted version of the LUCAS topsoil protocol (Tóth et al., 2013), modified to specifically target the rhizosphere of *D. purpurea*, with the aim of assessing microenvironmental differences potentially linked to distinct flower morphs. In each population, we selected nine individual plants (three per color morph: violet, pink, white) to sample soil at 15 cm depth using a soil‐corer. For each sample, we took nine subsamples: one central, taken directly beneath the plant base, and eight samples distributed equidistantly, collected along two perpendicular axes extending outward from the base, covering about a 1‐m buffer around the plant. All nine subsamples were pooled and thoroughly mixed, resulting in a single composite sample (approximately 1 kg) per plant. Soil variables were measured on this pooled mixture, providing one mean value per individual plant. For the native Swedish populations, we collected 27 (9 × 3) samples, but only 15 samples were sampled from the introduced populations in Bolivia because only two replicates per color morph were possible to sample in one of the populations. We calculated soil water content considering volume mass (wet vs. dry), soil pH, and total content of C, N, Fe, K, and P. All soils were analyzed at the Analytical Laboratory at Lund University.

### Statistical analyses

We fitted generalized linear models to assess the effects of flower color morph (violet, pink, white), population (B1, B2, G1, H1, H3), and their interaction on a set of response variables including plant size, floral traits, soil properties, pollinator visitation, and reproductive fitness using the function glm in the package stats in R version 4.4.3 (R Core Team, 2019). For each response variable, we constructed a series of models representing alternative hypotheses: (1) a null model (intercept only), (2) flower morph only, (3) population only, (4) flower morph + population [additive], and (5) flower morph × population [interaction]. Models were compared using the finite‐sample corrected Akaike information criterion (AICc), with the best‐supported models identified by the lowest AICc and highest AICc weight. Models within ΔAICc < 2 were considered equally plausible. We chose adequate error distributions depending on the type of response variable. Residual diagnostics for selected models (best or equally supported) were performed using the R package DHARMa (Hartig, 2022), which included checks for residual uniformity, dispersion, and model fit. Model selection was used to evaluate relative support among competing hypotheses and was not intended as a significance test of the null hypothesis; therefore, *P*‐values are not reported for AICc‐based model comparisons.

For flower traits and fitness, we included Plant ID as a random effect in addition to site origin to account for the repeated measurements within individual. Fruits, seeds, and germination could be calculated only for the Swedish populations and the B1 population in Bolivia due to the inaccessibility of the other population. Adjusted pollinator visitation rate was calculated based only on *Bombus* sp. visits because other groups such as bees (e.g., *Apis mellifera*, Halictidae) and Syrphidae were very rarely observed (~0.6%; Appendix S4) and all *Bombus* species observed belong to the same functional group of long‐tongued bumble bees. Adjusted pollinator visitation rate was log‐transformed to improve normality. In addition, we included Spearman correlation test between soil, plant size, and flower traits variables. Because most of the best models included population and flower color (see Table 1), we additionally tested the effect of color per population separately, we applied an ANOVA type II and pairwise comparisons with Tukey test using the R package emmeans version 2.0.2 (Lenth and Piaskowski, 2026).

**Table 1:** Model selection results assessing the effect of flower color (violet, pink, white), populations and its interaction on plant size, floral traits, pollinator visitation, reproductive fitness, and soil properties. For each response variable, we present either the best‐supported model (lowest AICc, highest weight) or models considered equally plausible (ΔAICc < 2). *k* = number of model parameters. Full model comparisons are provided in Appendix S5.

Model	Probability distribution	AICc	ΔAICc	*k*	Weight
**Plant size**					
m02: Rosette ~ Population	Gaussian	1695.7	0	6	0.56
m03: Rosette ~ Flower color + Population	Gaussian	1696.2	0.52	8	0.43
m02: Total height ~ Population	Gaussian	2133.2	0	6	0.87
**Flower traits**					
m03: Whole corolla size ~ Flower color + Population + (1|Plant_ID)	Gaussian	3850	0	9	0.66
m02: Whole corolla size ~ Population + (1|Plant_ID)	Gaussian	3851.8	1.8	7	0.26
m02: Prox. corolla size ~ Population + (1|Plant_ID)	Gaussian	2156.6	0	7	0.67
m03: Prox. corolla size ~ Flower color + Population + (1|Plant_ID)	Gaussian	2158.1	1.48	9	0.32
**Pollinators**					
m04: Visitation (adj) ~ Flower color * Population	Gamma (log)	377.3	0	15	0.99
**Reproductive fitness**					
m03: Fruits per plant ~ Flower color + Population + (1|Plant_ID)	Poisson	1344.3	0	7	0.74
m03: Seeds per fruit ~ Flower color + Population + (1|Plant_ID)	Negative binomial	10,704.6	0	8	0.79
m02: Germination ~ Population + (1|Plant_ID)	Binomial	1977.9	0	5	0.57
m03: Germination ~ Flower color + Population + (1|Plant_ID)	Binomial	1978.6	0.67	7	0.41
**Soil**					
m02: Water content ~ Population	Gaussian	295.1	0	6	0.93
m02: pH ~ Population	Gaussian	82.3	0	6	0.84
m02: C ~ Population	Gaussian	198.4	0	6	0.93
m02: N ~ Population	Gaussian	–81.7	0	6	0.94
m0: Fe ~ 1	Gaussian	310.6	0	2	0.85
m0: K ~ 1	Gaussian	119.2	0	2	0.76
m01: P ~ Flower color	Gaussian	–42.66	0	4	0.91

Finally, we tested whether among‐population differences in the frequencies of flower color morphs are associated with any variations in average soil conditions. Mean soil values were calculated for each population by averaging individual‐level measurements. We then tested for associations between population‐level soil variables and flower color morph frequencies using Spearman rank correlations including a false discovery rate (FDR) correction. Because soil variables are multivariate and potentially covary, we also performed a principal component analysis (PCA) on standardized population‐level soil variables to summarize overall soil profiles and to explore whether populations differing in flower color composition cluster in multivariate soil space. R version 4.4.3 was used for all analyses (R Core Team, 2019).

## RESULTS

We measured a total of 225 individuals from the five populations. For all the populations, except G1 in Sweden, we sampled almost all the individuals (Appendix S1). An overview table with means and standard deviations of plant size, flower traits, reproductive fitness and pollinator visitation rate (adjusted) per color morph and populations can be found in Appendix S6.

### Flower color

The petal reflectance spectra (Figure 1) clearly illustrated the relevance of our chosen color categories. In bee vision, the color morphs are similar in the UV range below the threshold given by a specular offset of *R*_spec_ ≈ 6% observed around 350 nm (due to the measurement at normal incidence), and in the long‐wavelength (red) range of diffuse reflectance seen around 700 nm (the long‐pass function equal to *R*_diff_/[1 + (*λ*_½_/*λ*)^*γ*^], *R*_diff_ ≈ 35%). The differences in reflectance between the morphs occurred in the range between these limits, defined by the long‐pass cut‐on flank, *λ*_½_ ≈ 420 nm and the steep slope, *γ* ≈ 17, which corresponds to the inflection point of the reflectance curve, a region of maximal spectral change that coincides with peak bee color discrimination sensitivity (Δ*λ*/*λ*) around 400 nm (Shrestha et al., 2013). Variation of the anthocyanin pigment with peak absorption around 550 nm accounting for the different shades of violet. Reflectance from petals was highest in white flowers, indicating low pigment absorption, and gradually decreased through light pink, dark pink, and violet morphs (Figure 1B).

It is noteworthy that the long‐pass cut‐on flank coincides with the blue spectral receptor of bee pollinators where they are highly sensitive to chromatic changes as opposed to the blue sensitivity in human color vision peaking around 445 nm. Because of this and the UV band, petals appearing white to humans display some 50% of chromatic saturation (Figure 1C). Under the *B. terrestris* vision model, bumblebees might be able to distinguish between all colors, and all color morphs displayed high contrast against green vegetation, with violet morphs having only slightly higher contrast than white petals (Figure 1C, insert 1D).

In contrast, the reflectance spectra of the inner nectar guide spots overlapped considerably, particularly between dark pink and violet flowers (Figure 2B). The reflectance minimum around 550 nm, was deepest in violet and dark pink flowers but overall, less pronounced than in the petal spectra. As a result, values in the bee color hexagon overlapped substantially, with no clear separation among flower morphs (Figure 2C). All points fell below the discrimination threshold of 9% hexagon units, indicating that the different color morphs are likely to be perceived as visually similar by bees (Figure 2D).

### Flower color‐morph frequency

Flower color‐morph frequencies differed clearly between regions. In Bolivia, violet flowers dominated (70–87%), with pink occurring at low proportions (13–17%) and no white individuals present in B1, while white reached 13% in B2. In contrast, the color morphs were more equally distributed in the Swedish populations. At G1, violet, pink, and white accounted for 27%, 43%, and 30% of individuals, respectively, while at H1, the proportions were 43% violet, 38% pink, and 19% white. At H3, pink was the dominant morph (69%), followed by violet (20%) and white (11%) (Appendices S1, S2).

Population‐level analyses using mean soil values did not reveal significant associations between the frequency of any color morph and individual soil variables, including nutrients, pH, and water content (Spearman correlations, all *P* > 0.05; all FDR‐adjusted *P* > 0.05). The PCA revealed a clear separation between Swedish and Bolivian populations along PC1, which explained 81.2% of the total soil variation and was primarily associated with soil water, nitrogen and carbon content (Appendix S7). In contrast, PC2 explained a smaller proportion of soil variation (11.8%) and was mainly associated with phosphorus, separating it from the other soil characteristics. Flower color‐morph frequencies did not align consistently with either PC axis or with any of the major soil gradients.

### Plant size and flower traits

Plant size (rosette diameter and total height) differed among populations (AICc = 1695.7, 2133.2; weight = 0.56, 0.87, respectively), but not among color morphs, although for rosette diameter adding flower color was also plausible (ΔAIC < 2, weight = 0.43; Table 1; Appendix S8). Whole and proximal corolla size were equally explained by population (AICc = 3850, 2158.1; weight = 0.66, 0.32) or population + flower color (weight = 0.26, 0.67; ΔAIC < 2, Table 1). On average, plant size traits did not differ between Bolivia and Sweden; although, Bolivian populations had smaller whole corollas but larger proximal corollas than those from Sweden. Within‐population analyses revealed no detectable differences in rosette diameter, total height, whole corolla, or proximal corolla among flower color morphs. However, in the Swedish population H1, pink plants were taller, and in the Bolivian population B2, violet flowers had smaller corollas (Appendices S8, S9).

Flowers from the Swedish populations showed no signs of nectar robbing, with the exception of a small proportion (9%) at H1 (*N* = 19; 12 violet, 6 pink, 1 white). In contrast, nectar robbing was much higher in the Bolivian populations, with 67% of flowers affected at B1 (*N* = 73; 65 violet, 8 pink) and 47% at B2 (*N* = 62; 42 violet, 10 pink, 10 white). Finally, plant size (rosette diameter and total height) was positively correlated with whole corolla size, but only weakly or not at all with proximal corolla size (Appendix S10A). Water content, carbon, and nitrogen from the soil were positively correlated to both plant and flower size traits (Appendix S10B).

### Pollinator observations

The most common pollinators of *D. purpurea* were *Bombus hortorum* and *B. pascuorum* in the Swedish populations and *B. funebris* in the Bolivian populations. In Bolivia, we also observed a few visits by *B. rubicundus* (pollinating and robbing) and by *Apis mellifera*, Halictidae, and Syrphidae (Appendix S4). Unexpectedly given previous observations in the B2 population (see Methods), no hummingbird visits were observed in any of the Bolivian populations.

**Figure 3:**
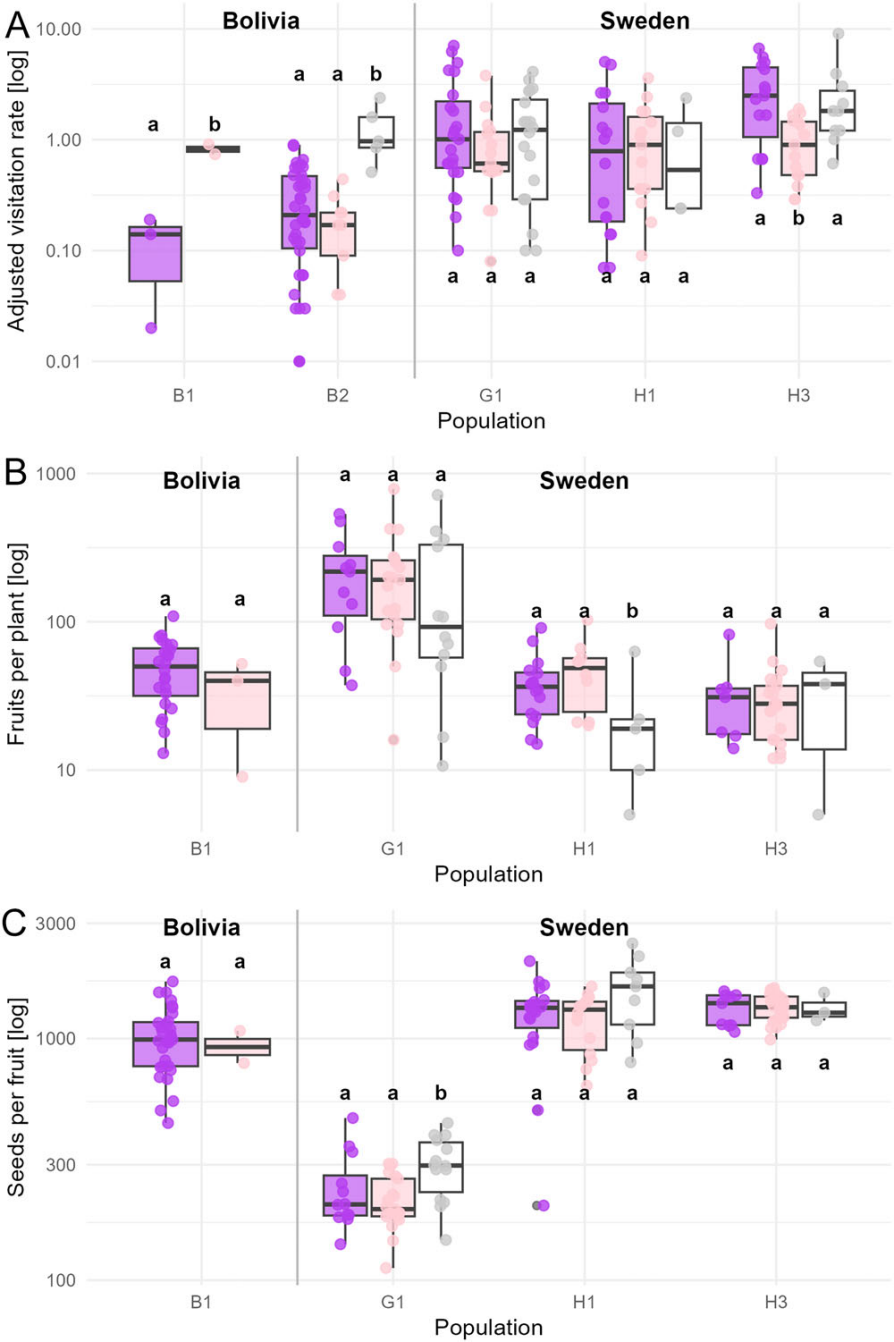
(A) Pollinator visitation rate, (B) fruits per plant, and (C) seeds per fruit across flower color morphs and populations in *Digitalis purpurea*. Flower color did not have a consistent effect on any trait, but all three variables varied among populations. Visitation was generally higher to Swedish populations than Bolivian, except for the white morph (A). Bolivian population B1 had fruit and seed set values similar to Swedish populations, whereas Swedish population G1 produced more fruits per plant (B) but fewer seeds per fruit (C). Different letters indicate significant differences based on Tukey post‐hoc tests within each population.

Adjusted *Bombus* visitation rate varied among morphs in a population‐specific way (full model with flower color × population interaction, AICc = 377.3; weight = 0.99, Table 1), with higher rates on average in the Swedish populations (Figure 3A). More specifically in Bolivia, pink flowers were visited more than violet flowers at B1; while white ones were more visited at B2. In Sweden, only H3 showed different visitation per color morph with pink ones being the least visited of the three (Figure 3A).

### Reproductive fitness

Fruits per plant and seeds per fruit varied among flower morphs and populations (AICc = 3740.6, 10,704.6; weight = 0.86, 0.79 respectively, Table 1). In the Bolivian B1 population, fruit and seed set did not differ between violet and pink morphs, and values were comparable to those observed in the Swedish populations H1 and H3. In the Swedish H1 population, white morphs produced fewer fruits per plant than violet and pink morphs, whereas in G1, white morphs had more seeds per fruit than the other two color morph (Figure 3B, C). Fruit production was five times higher in the largest population (G1, >500 individuals), with ~200 fruits/plant compared to 31, 45, and 47 fruits/plant at H3, H1, and B1, respectively (Figure 3B). In contrast, the number of seeds per fruit was lower at G1 (~200 seeds/fruit compared to >1000 seeds/fruit in all the other populations, Figure 3C).

Germination rates differed among populations (AICc = 1977.9; weight = 0.57) and color morphs (AICc = 1978.6; weight = 0.41; ΔAIC < 2, Table 1), but no differences were detected among morphs within populations (Appendix S9). On average, there was a lower germination rate for the Bolivian population compared to the Swedish populations (Appendix S11).

### Soil analysis

**Figure 4:**
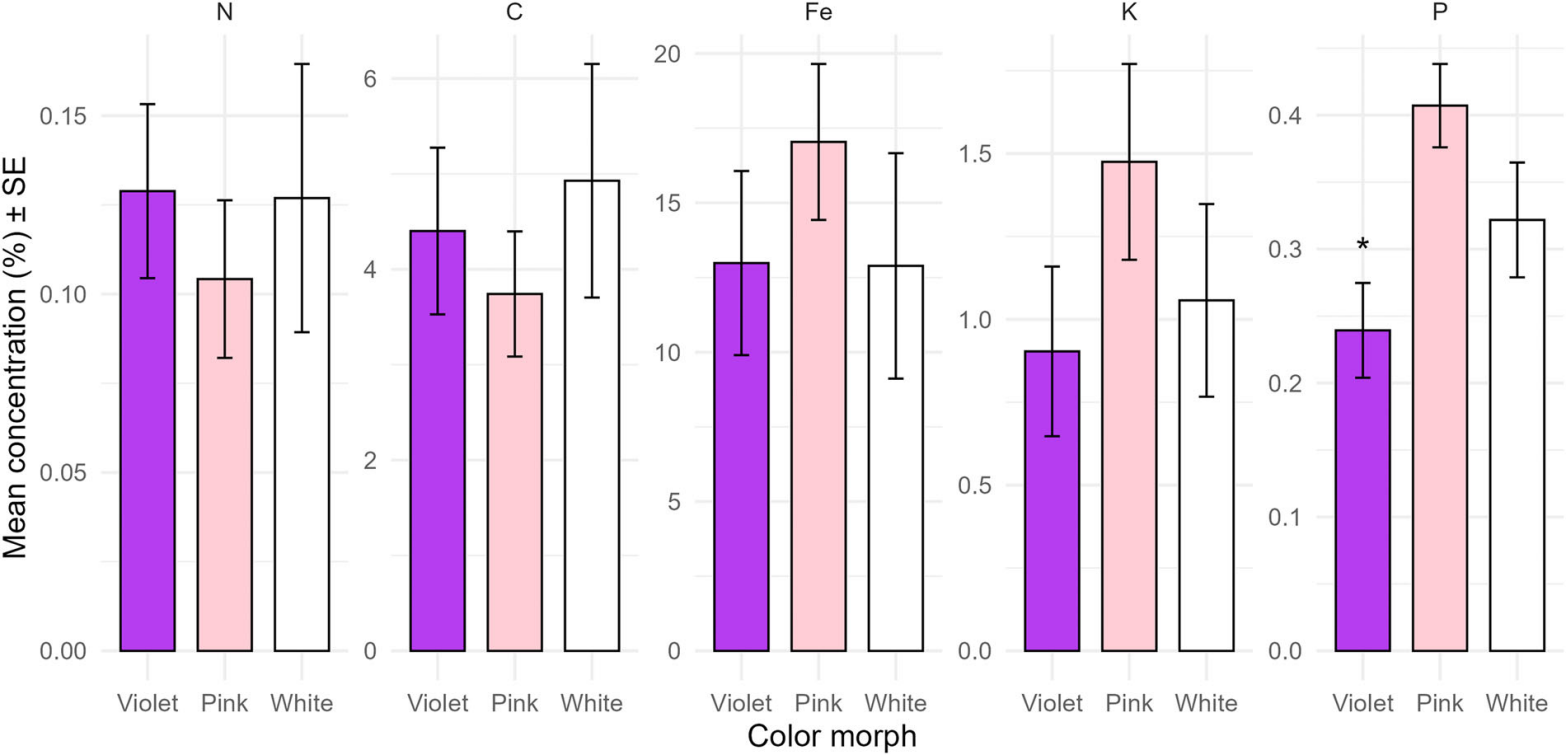
Soil nutrient concentrations for each flower color morph. Only phosphorus had an effect on flower color. Significance levels: *P < 0.05, **P < 0.01, ***P < 0.001. Note the different *y*‐axis scales.

Variation in soil water content, pH, and carbon and nitrogen concentrations was best explained by population identity (weights = 0.93, 0.84, 0.93, 0.94, and AICc = 295.1, 85.3, 198.4, −81.7 respectively; Table 1). In contrast, soil iron and potassium concentrations were not explained by any of the tested variables, with the null model providing the best fit (weights = 0.85 and 0.76, AICc = 310.6, 119.2 respectively; Table 1). Only the concentration of phosphorus in the soil differed among color morphs, with a lower concentration below violet individuals than below pink and white individuals (Table 1, Figure 4). In populations from Bolivia, pH values were around 6, while in Sweden values varied from 4 to 5 (Appendix S12A). Water content (%) was lower in the Bolivian populations and the Swedish G1 population (5‐10%) compared to 20–40% in H1 and H3 (Appendix S12B). Soil characteristics differed between Sweden and Bolivia, but they had no effect on flower color frequency at population level (see above).

## DISCUSSION

Understanding how floral trait variation influences pollination performance and reproductive success is key to evaluating the ecological and evolutionary trajectories of both native and introduced plant species. By combining data from both native populations in Sweden and introduced populations in Bolivia, we aimed to understand how both biotic (pollinators) and abiotic (soil characteristics) factors may influence the evolution of flower color variation in *Digitalis purpurea*.

Our findings suggest that flower color polymorphism is present across both regions but is not strongly shaped by either pollinator visitation or soil characteristics. Although introduced vs native populations varied in soil composition and soil phosphorus concentration differed among color morphs—with violet individuals associated with lower P levels—there was no evidence for consistent differences in pollinator visitation or reproductive output directly linked to flower color across sites. Instead, reproductive fitness components (fruit and seed production, germination) were more strongly influenced by population‐level variation, suggesting that unmeasured local ecological or demographic factors play a larger role. The limited visual discrimination among color morphs in bee color space, particularly due to overlapping nectar guide reflectance and lower chromatic contrast under bee vision, further supports the idea that flower color may not usually be subject to pollinator‐mediated selection in this system.

Reproductive fitness components were related to variation in flower color, but to a larger extent to variation in unmeasured site‐level factors. These results emphasize the context‐dependence of flower color expression and fitness in *Digitalis purpurea*, suggesting that its presence across native and introduced ranges may be shaped by site‐specific ecological interactions (such as herbivory, drought, UV exposure) rather than pollinators or soil conditions. Moreover, the persistence of flower color polymorphisms across diverse environments, despite the lack of detectable associations with the selective agents examined here, is consistent with the possibility that such variation may be selectively neutral or maintained by weak or context‐dependent selection.

### No evidence for consistent pollinator visits to flower color morphs

Although reflectance analysis of flower petals showed that bumblebees could potentially distinguish between all color morphs, we found no evidence of higher pollinator visits to a particular color morph across sites (Figures 1, 3A). Bumblebees, the dominant visitors at all sites, visited violet, pink, and white flowers at different rates when accounting for morph availability, depending on the population of origin. These results align with previous findings that *D. purpurea* pollinators do not exhibit strong color preferences (Ernst, 1987), likely because they respond to a broader suite of floral cues. Another possible explanation is that flower color could act as a visual attractant only when color morphs differ in floral rewards. However, to our knowledge, no studies have quantified nectar or pollen production among color morphs in *D. purpurea*, and such data would be valuable for testing this hypothesis.

Notably, our reflectance data showed no detectable differences in the pigmentation of the inner floral spots (nectary guides) among color morphs (Figure 2). The consistent presence and coloration of these nectar guides across morphs suggest that they may represent canalized traits maintained by stabilizing or directional selection. By providing reliable visual cues to pollinators, these traits may likely enhance reproductive success (Hansen et al., 2012; Zhang et al., 2017) and operate independently of variation in overall petal color or other floral traits. A similar pattern has been observed in *Iris petrana*, where the dark signal spot remains constant despite changes in flower size (Lozada‐Gobilard et al., 2022). The conserved nature of these spots in *Digitalis* may reflect their crucial role in pollinator guidance and suggest that selection on these guides may be decoupled from selection on petal color.

Nectar guides may also help reduce nectar robbing by promoting legitimate pollinator entry (Leonard et al., 2013), but this effect may be limited to the native range of *D. purpurea*. Nectar robbing appears to be rare in native populations and far more frequent in introduced ones. In our study, Swedish native populations showed low or no signs of nectar robbing (G1 = 0%, H1 = 9%, H3 = 0%), while it was widespread in the introduced Bolivian populations (B1 = 67%, B2 = 47%). A common garden experiment using native UK populations showed that nectar robbing reduced pollinator visitation, fruit set, and seed production (Mackin et al., 2021a). In contrast, our results suggest that fruit and seed set in the Bolivian populations were comparable to those of native populations with minimal nectar robbing (Figure 3), indicating that robbing may not strongly affect reproductive success in these introduced sites (Stout et al., 2000; Irwin et al., 2015). However, nectar robbing was not the primary focus of our study, and dedicated experiments and more extensive observations are needed to confirm this pattern.

In addition to flower color, other floral signals such as nectar guides and display size play important roles in attracting pollinators (van der Kooi et al., 2023). Indeed, Grindeland et al. (2005) found that pollinator visitation to *D. purpurea* increases with floral display size, suggesting that bumblebees are likely drawn to larger floral displays from a distance, and once on the plant, they might rely on consistent nectar guide patterns—rather than petal color—when foraging. This combination of visual cues may explain why visitation rates varied among flower color morphs in a population‐specific manner, rather than showing consistent patterns across sites (Table 1, Figure 3A). Given their angular resolution of ~1° (Land, 1997), bees can distinguish a 2 cm flower from about 1 m away but perceive the small nectar‐guide spots only within a few centimeters. Under natural conditions, petals are viewed under diffuse rather than specular light, so pollinators experience floral colors differently from our controlled reflectance measurements. Although we found no evidence of color‐based pollinator visitation rate—likely due to uniform pigmentation of inner spots across morphs—future studies should explore behavioral responses such as handling time to better understand how pollinators interact with floral color variation.

### Pollinator visitation did not explain geographical variation in plant fitness

Our study revealed higher variation in reproductive fitness among populations than flower color morphs, with white morphs producing fewer fruits in the Swedish population H1 but more seeds per fruit in the G1 population. However, these differences were not associated with pollinator visitation rates suggesting that geographical variation and factors other than pollinators may be shaping reproductive outcomes. In particular, the largest native population in Sweden (G1) exhibited substantially higher fruit production per plant, yet a markedly lower seed count per fruit. This inverse pattern may indicate reduced pollination efficiency, potentially resulting from demographic effects associated with high plant density (>500 individuals; Appendix S3). The reduced seed set per fruit observed in the largest and most densely populated *D. purpurea* site may indicate pollen limitation or inbreeding effects. Pollinators tend to visit more flowers overall on plants with larger floral displays, but also to visit a smaller proportion of the total flowers compared to plants with fewer flowers (Ohashi and Yahara, 1999; Harder et al., 2004), which may reduce effective cross‐pollination and lead to reduced seed set.

Germination rates were similar across flower color morphs, further supporting the idea that post‐pollination processes are decoupled from floral pigmentation. Notably, germination was consistently lower for the Bolivian population, possibly reflecting environmental constraints or inbreeding depression after a bottleneck effect caused by the invasion. However, this interpretation should be treated with caution; for a better understanding of these patterns, future studies should include reciprocal transplant experiments in natural habitats (Romero‐Bravo and Castellanos, 2024).

It is worth noting that Ernst (1987) reported reduced germination and seed production in white‐flowered *D. purpurea* individuals that likely represented albino mutants that lack anthocyanins. In contrast, the white morphs in our study usually retain pigmentation in the inner spots of the corolla, suggesting partial anthocyanin expression rather than complete albinism. This difference could indicate that the plants examined here represent a stable floral color polymorphism rather than albino variants, which may explain the discrepancies in reproductive performance reported between the two studies.

We acknowledge that reproductive fitness data could not be collected for one of the introduced Bolivian populations due to logistical constraints caused by dangerous heavy rains during the field campaigns. This limitation restricts our ability to fully compare fitness outcomes across all populations. However, the inclusion of other key traits—such as flower color distribution, floral reflectance, and pollinator visitation—still allows us to draw meaningful conclusions about the ecological and potential selective factors influencing color morphs in both native and introduced ranges.

### Soil phosphorus, but not other soil properties, explains variation in flower color

We detected a difference in soil phosphorus levels among flower color morphs, with violet morphs growing in soils with less phosphorus than in the soils with the pink and white morphs. While intriguing, this result should be interpreted cautiously because we found no consistent differences among the flower color morphs in other soil nutrients or pH. Instead, site explained more variation than flower color itself. Still, it is possible that soil nutrient availability could interact with pigment production pathways. It was previously found that higher phosphorus in the soil decreased anthocyanin accumulation producing lighter color hues (Kao, 1960; Chen et al., 2013), but at the same time high phosphorus enhance plant growth (Chen et al., 2013). Interestingly, overall, low phosphorus concentration was related to violet individuals (Figure 4), but pink and white ones were not larger than violet individuals in terms of rosette diameter or total height (Appendix S8). Although white‐flowered plants were predicted to experience nutrient stress, based on previous studies by Ernst (1987), we did not find evidence supporting this. Neither plant size nor floral morphology varied consistently among color morphs, suggesting that white morphs may not have a physiological disadvantage, at least at the sites sampled here.

In addition, plastic responses to abiotic conditions—such as soil ion concentrations and pH—may also influence flower coloration (Schreiber et al., 2011; Yoshida et al., 2020). Environmental effects can influence pigment expression, especially under nutrient, water, or pH stress (Warren and Mackenzie, 2001; Strauss and Whittall, 2006). Furthermore, soil moisture can modulate plant–animal interactions by influencing floral traits such as flower size or nectar production, which in turn may alter visitor behavior, potentially shifting interactions along a continuum from mutualism (e.g., pollination) to antagonism (e.g., nectar robbing) (Cha et al., 2025). In our study, soil water content and carbon and nitrogen concentrations were positively correlated with plant size and flower size (Appendix S10B), supporting the idea that resource availability shapes phenotypic traits relevant to pollinator interactions. Such context‐dependent dynamics may further obscure direct relationships between flower color and ecological function.

At the population level, additional analyses integrating mean soil properties across populations revealed that no single soil variable, including phosphorus, was consistently associated with variation in color morph frequencies. Spearman correlations and a principal component analysis of population‐level soil profiles showed differentiation among populations and between regions, but flower color frequencies did not align strongly with the major axes of soil variation (Appendix S7). It is important to note that these population‐level analyses are based on a small number of populations, limiting the scale of inference; therefore, the results should be interpreted cautiously, and additional populations should be studied to confirm these exploratory findings. Overall, these findings suggest that while individual‐level phosphorus variation may influence flower color locally, broader geographic patterns of color morph distribution are unlikely to be driven solely by soil nutrients.

## CONCLUSIONS

This study suggests a complex and context‐dependent nature of flower color variation in *Digitalis purpurea*. While floral color variation is probably genetically based and visually distinct to pollinators, we found little evidence of consistent pollinator visits to specific color morphs or uniform associations with soil characteristics across populations. Population‐level analyses further showed that average soil conditions, assessed using both univariate correlations and multivariate soil profiles, were not strongly associated with variation in flower color frequencies among populations, despite clear differences in soil characteristics between native and introduced ranges. Instead, variation in flower color, plant size, and floral traits appears to be shaped by local environmental conditions and geographic context. Reproductive fitness components—such as fruit and seed production—varied substantially among populations and, to a lesser extent, among color morphs. However, these differences were not aligned with pollinator visitation patterns, suggesting that factors other than pollinators, such as plant density, microhabitat variation, or abiotic stressors, are more likely drivers of reproductive success. Overall, these results emphasize the context dependency of flower color expression and fitness in *D. purpurea* and suggest that the observed flower color polymorphism could be neutral. The maintenance of multiple color morphs across a broad geographic range—despite differences in climate, soil, and pollinator communities—may therefore reflect a combination of weak selection, drift, and historical contingency, rather than directional or balancing selection by pollinators or abiotic constraints. These findings underscore the importance of considering both biotic and abiotic factors across spatial scales when investigating the maintenance of phenotypic diversity in wild plant populations. Future research integrating experimental, genetic, and long‐term ecological approaches will be essential to fully understand the evolutionary dynamics of floral color variation, especially in the context of global change and species introductions.

## AUTHOR CONTRIBUTIONS

S.L.G. and Ø.H.O. designed the study. S.L.G., P.P.E., G.P., Z.A., and E.L. collected data. M.B. contributed with the reflectance methods. S.L.G. analyzed the data and wrote the manuscript with help of Ø.H.O. and M.B. All co‐authors contributed significantly with helpful discussions and worked together on the final version of the manuscript.

## Supporting information


**Appendix S1.** Description of the sample populations.

**Appendix S2.** Location of the sampled populations and flower color proportions.

**Appendix S3.** Germination assay of *Digitalis purpurea*.

**Appendix S4.** List of pollinators visiting different flower color morphs in five populations of *Digitalis purpurea* in Bolivia (B1, B2) and Sweden (G1, H1, H3).

**Appendix S5.** All model selection results assessing the effect of flower color, populations, and interactions.

**Appendix S6.** Overview of plant, flower traits, pollinators (visitation rate adjusted), and fitness measurements per population and flower color.

**Appendix S7.** Relationship between soil variables and flower color frequency.

**Appendix S8.** Plant size and flower traits per flower color and population.

**Appendix S9.** Within‐population tests of the effect of flower color on plant size, floral traits, pollinator visitation, fitness and soil variables.

**Appendix S10.** Spearman correlations between soil and plant and flower traits.

**Appendix S11.** Germination rate per flower color and population.

**Appendix S12.** Soil pH and water content per population and flower color morph.

## Data Availability

The data sets generated and analyzed during the current study are available in the Figshare repository: https://doi.org/10.6084/m9.figshare.29095451.
