# Supplementary material for: Flower color variation in Digitalis purpurea: Pollination and soil influences across native and introduced populations
Source: Am J Bot. 2026 Apr 3;113(4):e70186. doi: 10.1002/ajb2.70186 (PMC13103626; doi:10.1002/ajb2.70186)
Supplement: Supplementary file 1 — Appendix S1. Description of the sample populations. [file AJB2-113-e70186-s008.docx]

**Appendix S1.** Description of the sample populations.

| Country | Pop | Latitude | Longitude | Altitude  (m a.s.l.) | *N* sampled individuals (proportion) | *N* total sampled (estimated) individuals |
| --- | --- | --- | --- | --- | --- | --- |
| Bolivia | B1 | 16°19′9.3″S | 67°56′39.5″W | 3475 | 35 (0.87) violet  5 (0.13) pink  0 (0) white | 40 (~50) |
|  | B2 | 16°18′41.4″S | 67°54′13.7″W | 3101 | 32 (0.70) violet  8 (0.17) pink  6 (0.13) white | 46 (~50) |
| Sweden | G1 | 55°35′33.2″N | 13°24′52.5″′ | 61 | 12 (0.27) violet  20 (0.43) pink  14 (0.30) white | 46 (>500) |
|  | H1 | 55°56´ 27.2″N | 13°36′10.7″E | 110 | 20 (0.43) violet  18 (0.38) pink  9 (0.19) white | 47 (~50) |
|  | H3 | 55°54′39.9″N | 13°30′40.87″E | 80 | 9 (0.20) violet  32 (0.69) pink  5 (0.11) white | 46 (~50) |
