# Supplementary material for: Flower color variation in Digitalis purpurea: Pollination and soil influences across native and introduced populations
Source: Am J Bot. 2026 Apr 3;113(4):e70186. doi: 10.1002/ajb2.70186 (PMC13103626; doi:10.1002/ajb2.70186)

**Appendix S2.** Location of sampled populations and proportion of flower colors in the populations. Maps show the location of the sampled populations: two introduced populations B1 and B2 in Bolivia (upper) and three native, G1, H1, H3 in Sweden (bottom). Pie charts display the proportion of the individuals corresponding to each flower color: violet, pink and white (shown in gray).


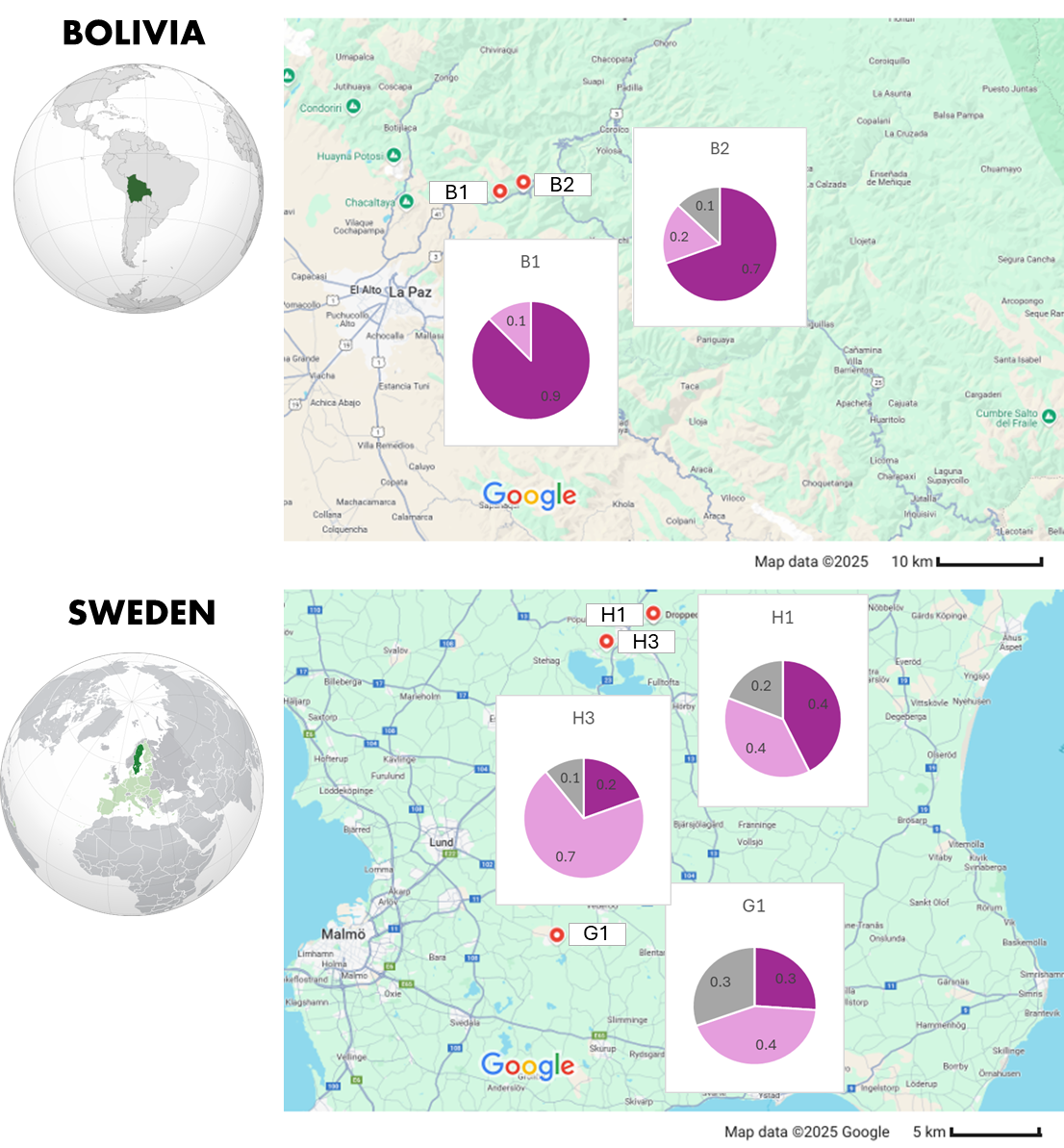

Supplement: Supplementary file 2 — Appendix S2. Location of the sampled populations and flower color proportions. [file AJB2-113-e70186-s009.docx]
