# Supplementary material for: Flower color variation in Digitalis purpurea: Pollination and soil influences across native and introduced populations
Source: Am J Bot. 2026 Apr 3;113(4):e70186. doi: 10.1002/ajb2.70186 (PMC13103626; doi:10.1002/ajb2.70186)
Supplement: Supplementary file 3 — Appendix S3. Germination assay of Digitalis purpurea. [file AJB2-113-e70186-s006.docx]

**Appendix S3.** Germination assay of *Digitalis purpurea* seeds from populations B1 (Bolivia), and G1, H1, and H3 (Sweden). The B1 population did not have any white individuals; Swedish populations had all three color morphs (i.e., white, pink, and violet). (A) Overview of experiment in the greenhouse. (B) Experimental set-up for the germination assay. We randomly selected 1–3 fruits per individual and sowed 30 seeds per fruit in peat-free soil and vermiculite in 0.5-mL pots; pots were regularly watered. Seed viability was evaluated for 25 days after sowing.

**
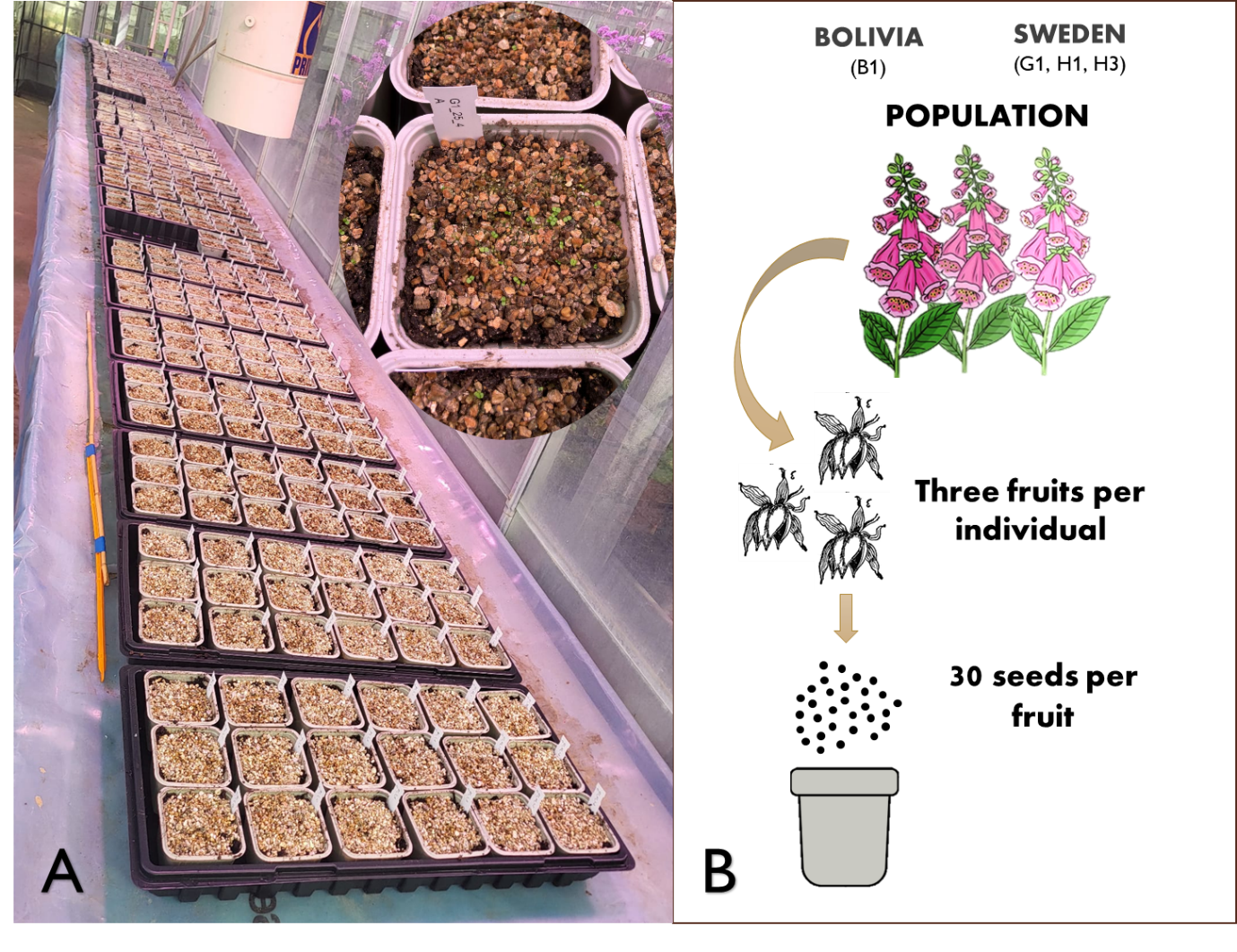
**
