# Supplementary material for: Flower color variation in Digitalis purpurea: Pollination and soil influences across native and introduced populations
Source: Am J Bot. 2026 Apr 3;113(4):e70186. doi: 10.1002/ajb2.70186 (PMC13103626; doi:10.1002/ajb2.70186)
Supplement: Supplementary file 4 — Appendix S4. List of pollinators visiting different flower color morphs in five populations of Digitalis purpurea in Bolivia (B1, B2) and Sweden (G1, H1, H3). [file AJB2-113-e70186-s011.docx]

**Appendix S4.** List of pollinators visiting different flower color morphs in five populations of *Digitalis purpurea* in Bolivia (B1, B2) and Sweden (G1, H1, H3).

| Pollinator groups | B1 |  | B2 |  |  | G1 |  |  | H1 |  |  | H3 |  |  |
| --- | --- | --- | --- | --- | --- | --- | --- | --- | --- | --- | --- | --- | --- | --- |
|  | **V** | **P** | **V** | **P** | **W** | **V** | **P** | **W** | **V** | **P** | **W** | **V** | **P** | **W** |
| Bees |  |  |  |  |  |  |  |  |  |  |  |  |  |  |
| *Andrena_sp* |  |  |  |  |  |  |  |  | 2 |  | 1 |  |  |  |
| *Apis_mellifera* |  |  | 14 |  |  |  |  |  |  |  |  |  |  |  |
| Halictidae | 2 |  | 1 |  |  |  |  |  |  |  |  |  |  |  |
| Birds |  |  |  |  |  |  |  |  |  |  |  |  |  |  |
| *Colibri_sp* |  |  | 15 |  |  |  |  |  |  |  |  |  |  |  |
| Bumblebess |  |  |  |  |  |  |  |  |  |  |  |  |  |  |
| *Bombus_cf_funebris* |  |  | 72 | 4 |  |  |  |  |  |  |  |  |  |  |
| *Bombus_funebris* | 23 |  | 990 | 35 | 111 |  |  |  |  |  |  |  |  |  |
| *Bombus_hortorum* |  |  |  |  |  | 226 | 52 | 26 | 180 | 116 | 17 | 44 | 32 | 15 |
| *Bombus_pascuorum* |  |  |  |  |  | 155 | 109 | 111 | 62 | 20 |  | 119 | 104 | 32 |
| *Bombus_rubicundus* | 36 | 32 | 13 |  |  |  |  |  |  |  |  |  |  |  |
| *Bombus_sp* |  |  |  |  |  | 14 | 5 | 46 | 3 | 1 |  | 17 | 27 | 2 |
| *Bombus_sp_black* |  |  |  |  |  | 4 |  | 3 |  |  |  |  |  |  |
| *Bombus_sp2* |  |  |  |  |  | 10 | 8 | 6 |  |  |  |  |  |  |
| Flies |  |  |  |  |  |  |  |  |  |  |  |  |  |  |
| Diptera |  |  |  |  |  |  |  |  | 2 |  |  |  |  |  |
| Syrphidae |  |  | 7 |  | 3 |  |  |  | 6 | 3 |  |  |  |  |
| Honeybees |  |  |  |  |  |  |  |  |  |  |  |  |  |  |
| *Apis_mellifera* |  |  | 11 | 3 |  | 1 |  |  | 23 | 9 |  | 5 |  |  |
| Total | **61** | **32** | **1123** | **42** | **114** | **410** | **174** | **192** | **278** | **149** | **18** | **185** | **163** | **49** |
