# Supplementary material for: Flower color variation in Digitalis purpurea: Pollination and soil influences across native and introduced populations
Source: Am J Bot. 2026 Apr 3;113(4):e70186. doi: 10.1002/ajb2.70186 (PMC13103626; doi:10.1002/ajb2.70186)
Supplement: Supplementary file 5 — Appendix S5. All model selection results assessing the effect of flower color, populations, and interactions. [file AJB2-113-e70186-s002.docx]

**Appendix S5.** All model selection results assessing the effect of flower color (violet, pink, white), populations and its interaction on (A) soil properties, (B) plant size, (C) floral traits, (D) pollinator visitation, and (E) reproductive fitness. In bold for each response variable is either the best-supported model (lowest AICc, highest weight) or the models considered equally plausible (ΔAICc < 2).

| Model | Probability distribution | df | AICc | dAICc | Weight |
| --- | --- | --- | --- | --- | --- |
| 1. Soil |  |  |  |  |  |
| m02: Water content ~ Population | **Gaussian** | **6** | **295.12** | **0.00** | **0.93** |
| m03: Water content ~ Flower color + Population | Gaussian | 8 | 300.23 | 5.11 | 0.07 |
| m04: Water content ~ Flower color * Population | Gaussian | 16 | 332.82 | 37.70 | 0.00 |
| m0: Water content ~ 1 | Gaussian | 2 | 338.17 | 43.05 | 0.00 |
| m01: Water content ~ Flower color | Gaussian | 4 | 342.45 | 47.33 | 0.00 |
| m02: pH ~ Population | **Gaussian** | **6** | **82.27** | **0.00** | **0.84** |
| m03: pH ~ Flower color + Population | Gaussian | 8 | 85.58 | 3.30 | 0.16 |
| m0: pH ~ 1 | Gaussian | 2 | 113.21 | 30.93 | 0.00 |
| m04: pH ~ Flower color * Population | Gaussian | 16 | 116.57 | 34.30 | 0.00 |
| m01: pH ~ Flower color | Gaussian | 4 | 117.52 | 35.25 | 0.00 |
| m02: C ~ Population | Gaussian | **6** | **198.36** | **0.00** | **0.93** |
| m03: C ~ Flower color + Population | Gaussian | 8 | 203.58 | 5.22 | 0.07 |
| m0: C ~ 1 | Gaussian | 2 | 211.16 | 12.80 | 0.00 |
| m04: C ~ Flower color * Population | Gaussian | 4 | 215.11 | 16.75 | 0.00 |
| m01: C ~ Flower color | Gaussian | 16 | 234.29 | 35.93 | 0.00 |
| m02: N ~ Population | **Gaussian** | **6** | **-81.75** | **0.00** | **0.94** |
| m03: N ~ Flower color + Population | Gaussian | 8 | -76.22 | 5.53 | 0.06 |
| m0: N ~ 1 | Gaussian | 2 | -67.97 | 13.78 | 0.00 |
| m04: N ~ Flower color * Population | Gaussian | 4 | -63.69 | 18.06 | 0.00 |
| m01: N ~ Flower color | Gaussian | 16 | -49.17 | 32.57 | 0.00 |
| m0: Fe ~ 1 | **Gaussian** | **2** | **310.60** | **0.00** | **0.85** |
| m01: Fe ~ Flower color | Gaussian | 4 | 314.16 | 3.57 | 0.14 |
| m02: Fe ~ Population | Gaussian | 6 | 319.99 | 9.39 | 0.01 |
| m03: Fe ~ Flower color + Population | Gaussian | 8 | 324.83 | 14.24 | 0.00 |
| m04: Fe ~ Flower color * Population | Gaussian | 16 | 340.72 | 30.13 | 0.00 |
| m0: K ~ 1 | Gaussian | **2** | **119.22** | **0.00** | **0.76** |
| m01: K ~ Flower color | Gaussian | 4 | 121.57 | 2.34 | 0.23 |
| m02: K ~ Population | Gaussian | 6 | 128.18 | 8.95 | 0.01 |
| m03: K ~ Flower color + Population | Gaussian | 8 | 131.89 | 12.66 | 0.00 |
| m04: K ~ Flower color * Population | Gaussian | 16 | 151.95 | 32.72 | 0.00 |
| m01: P ~ Flower color | **Gaussian** | **4** | **-42.66** | **0.00** | **0.91** |
| m03: P ~ Flower color + Population | Gaussian | 8 | -36.55 | 6.11 | 0.04 |
| m0: P ~ 1 | Gaussian | 2 | -36.44 | 6.23 | 0.04 |
| m02: P ~ Population | Gaussian | 6 | -30.16 | 12.50 | 0.00 |
| m04:P ~ Flower color * Population | Gaussian | 16 | -13.43 | 29.24 | 0.00 |
| 1. Plant size |  |  |  |  |  |
| m02: Rosette ~ Population | **Gaussian** | **6** | **1695.66** | **0.00** | **0.560** |
| m03: Rosette ~ Flower color + Population | **Gaussian** | **8** | **1696.18** | **0.52** | **0.432** |
| m04: Rosette ~ Flower color * Population | Gaussian | 15 | 1704.02 | 8.36 | 0.009 |
| m0: Rosette ~ 1 | Gaussian | 2 | 1780.28 | 84.63 | 0.000 |
| m01: Rosette ~ Flower color | Gaussian | 4 | 1780.44 | 84.78 | 0.000 |
| m02: Total height ~ Population | **Gaussian** | **6** | **2133.23** | **0.00** | **0.869** |
| m03: Total height ~ Flower color + Population | Gaussian | 8 | 2137.17 | 3.94 | 0.122 |
| m04: Total height ~ Flower color * Population | Gaussian | 15 | 2142.36 | 9.13 | 0.009 |
| m0: Total height ~ 1 | Gaussian | 2 | 2161.83 | 28.60 | 0.000 |
| m01: Total height ~ Flower color | Gaussian | 4 | 2164.80 | 31.57 | 0.000 |
| 1. Flower traits |  |  |  |  |  |
| m03: Whole corolla size ~ Flower color + Population + (1\|Plant_ID) | **Gaussian** | **9** | **3849.97** | **0.00** | **0.656** |
| m02: Whole corolla size ~ Population + (1\|Plant_ID) | **Gaussian** | **7** | **3851.78** | **1.81** | **0.265** |
| m04: Whole corolla size ~ Flower color * Population + (1\|Plant_ID) | Gaussian | 16 | 3854.21 | 4.23 | 0.079 |
| m01: Whole corolla size ~ Flower color + (1\|Plant_ID) | Gaussian | 5 | 3967.20 | 117.23 | 0.000 |
| m0: Whole corolla size ~ 1 + (1\|Plant_ID) | Gaussian | 3 | 3991.17 | 141.20 | 0.000 |
| m02: Prox. corolla size ~ Population + (1\|Plant_ID) | **Gaussian** | **7** | **2156.59** | **0.00** | **0.675** |
| m03: Prox. corolla size ~ Flower color + Population + (1\|Plant_ID) | **Gaussian** | **9** | **2158.06** | **1.48** | **0.322** |
| m04: Prox. corolla size ~ Flower color * Population + (1\|Plant_ID) | Gaussian | 16 | 2167.42 | 10.84 | 0.003 |
| m01: Prox. corolla size ~ Flower color + (1\|Plant_ID) | Gaussian | 5 | 2203.32 | 46.74 | 0.000 |
| m0: Prox. corolla size ~ 1 + (1\|Plant_ID) | Gaussian | 3 | 2214.87 | 58.29 | 0.000 |
| 1. Pollinators |  |  |  |  |  |
| m04: Visitation (adj) ~ Flower color * Population | **Gamma (log)** | **15** | **377.31** | **0.00** | **0.996** |
| m03: Visitation (adj) ~ Flower color + Population | Gamma (log) | 8 | 388.64 | 11.33 | 0.003 |
| m02: Visitation (adj) ~ Population | Gamma (log) | 6 | 397.08 | 19.77 | 0.000 |
| m01: Visitation (adj) ~ Flower color | Gamma (log) | 4 | 451.83 | 74.51 | 0.000 |
| m0: Visitation (adj) ~ 1 | Gamma (log) | 2 | 455.63 | 78.31 | 0.000 |
| 1. Reproductive fitness |  |  |  |  |  |
| m03: Fruits per plant ~ Flower color + Population + (1\|Plant_ID) | **Poisson** | **7** | **1344.33** | **0.00** | **0.73** |
| m02: Fruits per plant ~ Population + (1\|Plant_ID) | Poisson | 5 | 1346.52 | 2.18 | 0.25 |
| m04: Fruits per plant ~ Flower color * Population + (1\|Plant_ID) | Poisson | 12 | 1353.31 | 8.98 | 0.008 |
| m0: Fruits per plant ~ 1 + (1\|Plant_ID) | Poisson | 2 | 1424.53 | 80.19 | 0.00 |
| m01: Fruits per plant ~ Flower color + (1\|Plant_ID) | Poisson | 4 | 1428.47 | 84.13 | 0.00 |
| m03: Seeds per fruit ~ Flower color + Population + (1\|Plant_ID) | **Negative binomial** | **8** | **10704.61** | **0.00** | **0.79** |
| m02: Seeds per plant ~ Population + (1\|Plant_ID) | Negative binomial | 6 | 10707.44 | 2.83 | 0.19 |
| m04: Seeds per plant ~ Flower color * Population + (1\|Plant_ID) | Negative binomial | 13 | 10712.93 | 8.32 | 0.01 |
| m0: Seeds per plant ~ 1 + (1\|Plant_ID) | Negative binomial | 3 | 10962.72 | 258.11 | 0.00 |
| m01: Seeds per plant ~ Flower color + (1\|Plant_ID) | Negative binomial | 5 | 10963.37 | 258.76 | 0.00 |
| m02: Germination ~ Population + (1\|Plant_ID) | **Binomial** | **5** | **1977.92** | **0.00** | **0.572** |
| m03: Germination ~ Flower color + Population + (1\|Plant_ID) | **Binomial** | **7** | **1978.59** | **0.67** | **0.410** |
| m04: Germination ~ Flower color * Population + (1\|Plant_ID) | Binomial | 12 | 1984.86 | 6.94 | 0.018 |
| m01: Germination ~ Flower color + (1\|Plant_ID) | Binomial | 4 | 2057.75 | 79.83 | 0.000 |
| m0: Germination ~ 1 + (1\|Plant_ID) | Binomial | 2 | 2066.40 | 88.48 | 0.000 |
