# Supplementary material for: Flower color variation in Digitalis purpurea: Pollination and soil influences across native and introduced populations
Source: Am J Bot. 2026 Apr 3;113(4):e70186. doi: 10.1002/ajb2.70186 (PMC13103626; doi:10.1002/ajb2.70186)
Supplement: Supplementary file 6 — Appendix S6. Overview of plant, flower traits, pollinators (visitation rate adjusted), and fitness measurements per population and flower color. [file AJB2-113-e70186-s004.docx]

**Appendix S6.** Overview table of plant, flower traits, pollinators (visitation rate adjusted), and fitness measurements per population and flower color.

| Trait | Pop | Violet | | | Pink | | | White | | |
| --- | --- | --- | --- | --- | --- | --- | --- | --- | --- | --- |
|  |  | *N* | Mean | SD | *N* | Mean | SD | *N* | Mean | SD |
| Diameter_rosette | B1 | 35 | 50.41 | 11.11 | 5 | 45.80 | 15.45 |  |  |  |
|  | B2 | 32 | 37.09 | 13.62 | 7 | 33.57 | 15.77 | 6 | 31.83 | 8.95 |
|  | G1 | 11 | 36.55 | 6.31 | 20 | 30.88 | 5.36 | 14 | 34.31 | 8.13 |
|  | H1 | 20 | 49.07 | 10.16 | 18 | 51.13 | 10.02 | 9 | 45.06 | 11.09 |
|  | H3 | 9 | 55.11 | 10.36 | 32 | 49.49 | 11.49 | 4 | 60.25 | 2.36 |
| Height_total | B1 | 35 | 81.71 | 35.15 | 5 | 86.00 | 50.26 |  |  |  |
|  | B2 | 32 | 117.72 | 37.31 | 8 | 98.63 | 29.45 | 6 | 106.33 | 24.95 |
|  | G1 | 11 | 85.24 | 26.79 | 20 | 81.20 | 26.03 | 14 | 81.92 | 25.94 |
|  | H1 | 20 | 97.91 | 31.40 | 18 | 116.58 | 20.02 | 9 | 92.17 | 18.00 |
|  | H3 | 9 | 92.06 | 16.99 | 31 | 84.48 | 23.92 | 5 | 91.60 | 15.61 |
| Proximal_size | B1 | 33 | 6.98 | 1.05 | 5 | 7.13 | 1.37 |  |  |  |
|  | B2 | 31 | 7.68 | 0.73 | 7 | 7.92 | 0.72 | 6 | 7.71 | 0.83 |
|  | G1 | 12 | 6.71 | 0.50 | 20 | 6.69 | 0.51 | 14 | 6.86 | 0.50 |
|  | H1 | 19 | 7.23 | 0.37 | 18 | 7.25 | 0.43 | 9 | 7.39 | 0.67 |
|  | H3 | 8 | 6.96 | 0.53 | 29 | 6.67 | 0.74 | 4 | 6.95 | 0.61 |
| WholeCor_size | B1 | 33 | 20.54 | 2.10 | 5 | 20.68 | 3.78 |  |  |  |
|  | B2 | 31 | 23.10 | 1.54 | 7 | 24.85 | 2.26 | 6 | 24.67 | 1.77 |
|  | G1 | 12 | 23.14 | 1.26 | 20 | 23.97 | 1.93 | 14 | 24.26 | 1.67 |
|  | H1 | 19 | 25.60 | 1.89 | 18 | 25.53 | 1.41 | 9 | 25.97 | 2.39 |
|  | H3 | 8 | 25.27 | 2.78 | 29 | 24.89 | 2.17 | 4 | 26.17 | 0.86 |
| Pollinators (visitation rate adjusted) | B1 | 4 | 0.09 | 0.08 | 2 | 0.82 | 0.12 |  |  |  |
|  | B2 | 46 | 0.25 | 0.24 | 10 | 0.18 | 0.12 | 6 | 1.08 | 0.8 |
|  | G1 | 24 | 1.86 | 2.03 | 21 | 0.96 | 0.93 | 24 | 1.41 | 1.23 |
|  | H1 | 22 | 1.10 | 1.47 | 19 | 0.92 | 0.93 | 5 | 0.85 | 0.94 |
|  | H3 | 16 | 2.76 | 1.95 | 15 | 0.98 | 0.57 | 10 | 2.59 | 2.49 |
| Fruits per plant | B1 | 37 | 48.25 | 23.93 | 5 | 33.67 | 22.19 |  |  |  |
|  | G1 | 39 | 212.39 | 159.00 | 58 | 212.22 | 196.66 | 33 | 202.21 | 217.95 |
|  | H1 | 34 | 45.96 | 25.43 | 26 | 52.80 | 33.30 | 10 | 37.38 | 29.13 |
|  | H3 | 10 | 31.22 | 20.60 | 37 | 32.38 | 23.61 | 6 | 32.33 | 24.99 |
| Seeds per fruit | B1 | 91 | 1019.76 | 380.98 | 4 | 933.50 | 431.44 |  |  |  |
|  | G1 | 63 | 244.90 | 178.45 | 96 | 215.30 | 101.66 | 65 | 314.31 | 161.44 |
|  | H1 | 116 | 1318.72 | 637.84 | 89 | 1260.58 | 576.73 | 40 | 1351.30 | 683.57 |
|  | H3 | 46 | 1338.63 | 311.60 | 116 | 1348.31 | 308.23 | 12 | 1377.42 | 358.56 |
