# Supplementary material for: Flower color variation in Digitalis purpurea: Pollination and soil influences across native and introduced populations
Source: Am J Bot. 2026 Apr 3;113(4):e70186. doi: 10.1002/ajb2.70186 (PMC13103626; doi:10.1002/ajb2.70186)
Supplement: Supplementary file 7 — Appendix S7. Relationship between soil variables and flower color frequency. [file AJB2-113-e70186-s007.docx]

**Appendix S7.** Principal component analysis of relationship between soil variables and each flower color morph (A–C) in the four populations of *Digitalis purpurea*. Green points: native Swedish populations; red points, introduced Bolivian populations). Point size indicates the proportion of (A) white, (B) pink and (C) violet-flowered individuals. Arrows show soil variable loadings. Percentage variance explained by each axis is given in parentheses.


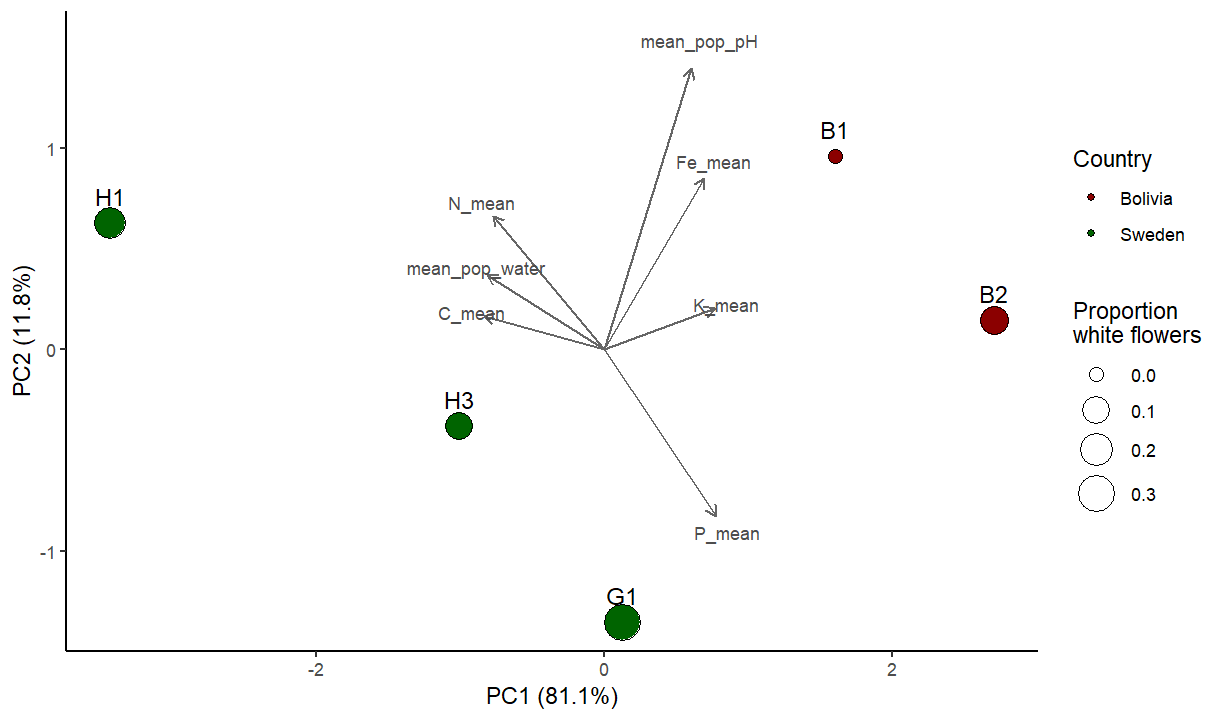


**A**


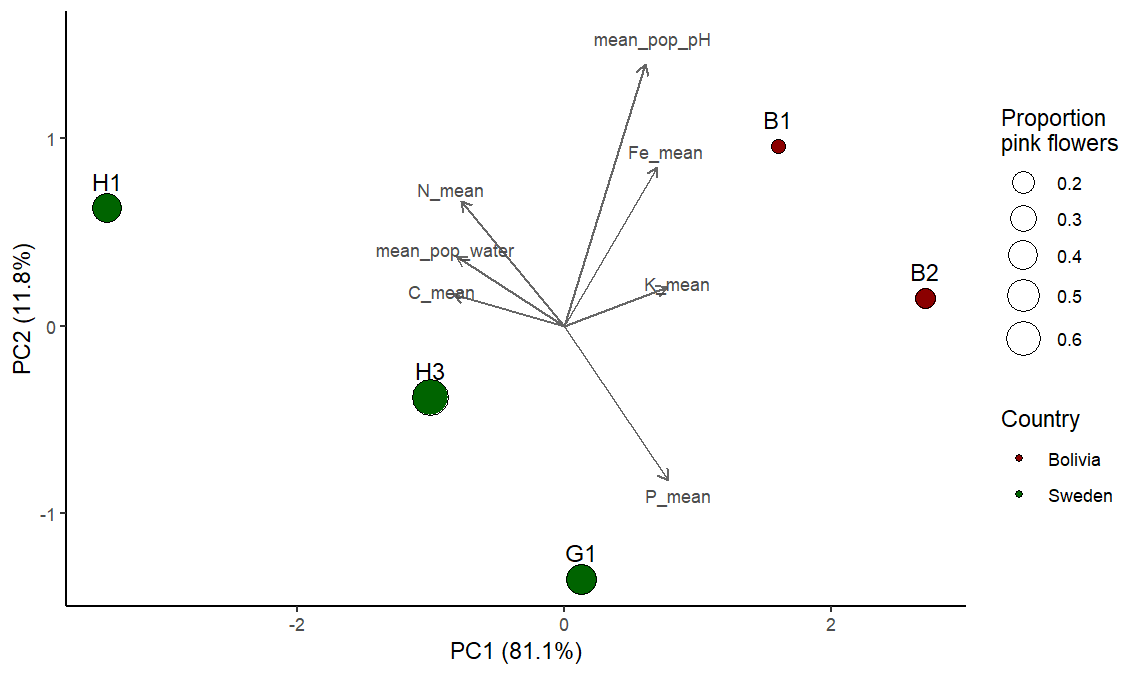


**B**


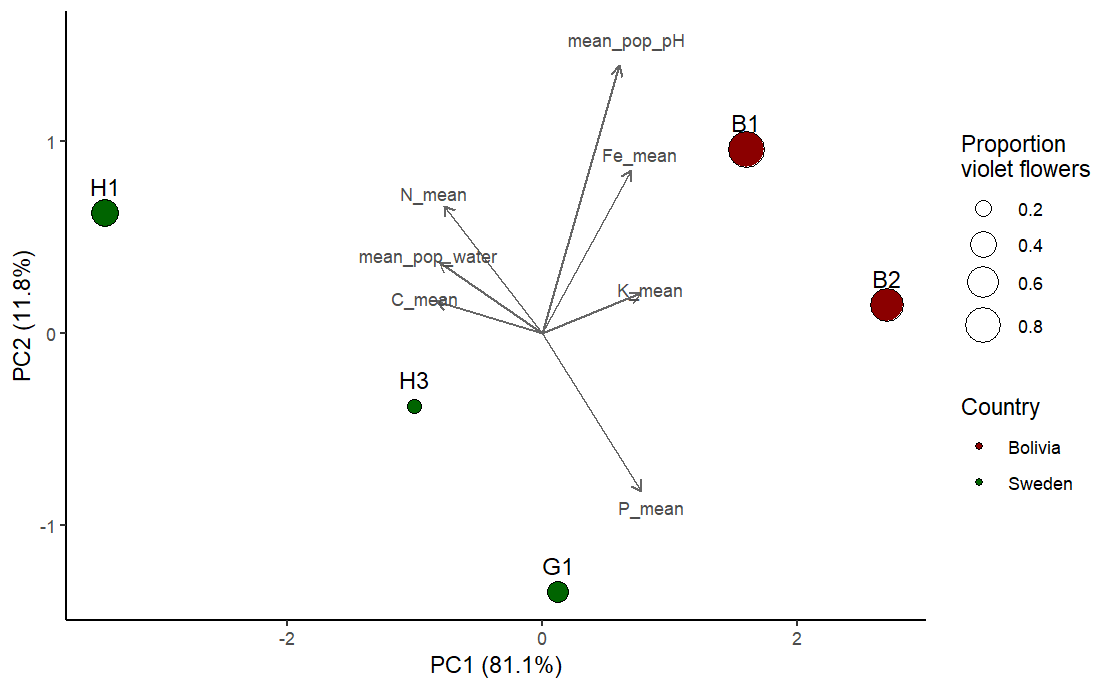


**C**
