# Supplementary material for: Flower color variation in Digitalis purpurea: Pollination and soil influences across native and introduced populations
Source: Am J Bot. 2026 Apr 3;113(4):e70186. doi: 10.1002/ajb2.70186 (PMC13103626; doi:10.1002/ajb2.70186)

**Appendix S8.** Plant dimensions and flower size in relation to flower color morph (violet, pink, white) and population (introduced Bolivian, Swedish) of *Digitalis purpurea*. Flower color alone did not affect (A) rosette diameter or (D) proximal corolla size. Flower color did not affect (B) total height in Bolivia, but pink individuals were larger in one population in Sweden. Whole corolla size (C) did not change by color in Sweden, but violet individuals were smaller in one of the two Bolivian populations.


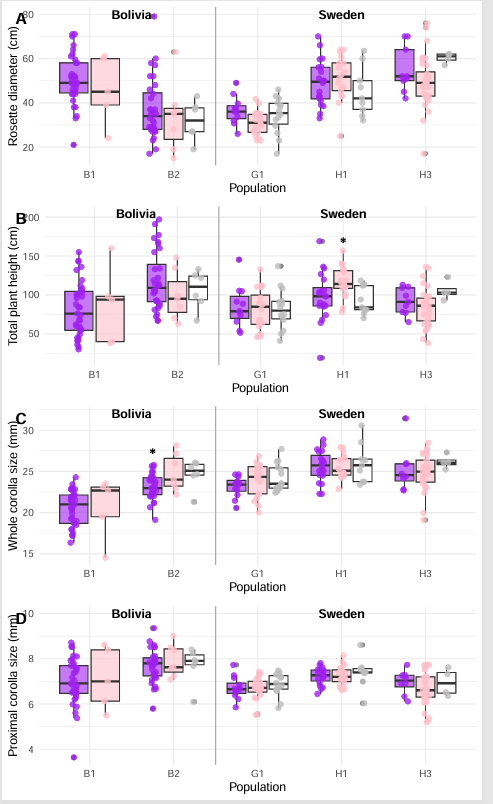

Supplement: Supplementary file 8 — Appendix S8. Plant size and flower traits per flower color and population. [file AJB2-113-e70186-s001.docx]
