# Supplementary material for: Flower color variation in Digitalis purpurea: Pollination and soil influences across native and introduced populations
Source: Am J Bot. 2026 Apr 3;113(4):e70186. doi: 10.1002/ajb2.70186 (PMC13103626; doi:10.1002/ajb2.70186)
Supplement: Supplementary file 9 — Appendix S9. Within‐population tests of the effect of flower color on plant size, floral traits, pollinator visitation, fitness and soil variables. [file AJB2-113-e70186-s010.docx]

**Appendix S9.** Within-population ANOVAs of the effect of flower color on plant size, floral traits, pollinator visitation, reproductive fitness, and soil variables. Generalized linear models (GLMs) were run separately for each population to assess the effect of flower color. Post hoc Tukey tests were used for pairwise comparisons between color morphs within a population; different letters indicate a significant difference in the trait between morphs (*P* < 0.05).

| Trait | Pop | ANOVA | | | Tukey comparisons | | |
| --- | --- | --- | --- | --- | --- | --- | --- |
|  |  | *χ*^2^ | df | *P* | Violet | Pink | White |
| Diameter_rosette | B1 | 0.72 | 1 | 0.395 | a | a |  |
|  | B2 | 1.08 | 2 | 0.584 | a | a | a |
|  | G1 | 6.17 | 2 | 0.046 | a | a | a |
|  | H1 | 2.23 | 2 | 0.327 | a | a | a |
|  | H3 | 5 | 2 | 0.082 | a | a | a |
| Height_total | B1 | 0.06 | 1 | 0.804 | a | a |  |
|  | B2 | 1.97 | 2 | 0.373 | a | a | a |
|  | G1 | 0.19 | 2 | 0.910 | a | a | a |
|  | **H1** | **8.1** | **2** | **0.017** | **a** | **b** | **a** |
|  | H3 | 3.88 | 2 | 0.144 | a | a | a |
| Proximal_size | B1 | 0.09 | 1 | 0.768 | a | a |  |
|  | B2 | 0.61 | 2 | 0.736 | a | a | a |
|  | G1 | 1.18 | 2 | 0.554 | a | a | a |
|  | H1 | 0.79 | 2 | 0.673 | a | a | a |
|  | H3 | 1.57 | 2 | 0.456 | a | a | a |
| WholeCor_size | B1 | 0.05 | 1 | 0.823 | a | a |  |
|  | **B2** | **9.56** | **2** | **0.008** | **a** | **b** | **b** |
|  | G1 | 3.22 | 2 | 0.199 | a | a | a |
|  | H1 | 0.3 | 2 | 0.863 | a | a | a |
|  | H3 | 1.13 | 2 | 0.569 | a | a | a |
| Pollinator visitation rate adjusted | **B1** | **11.1** | **1** | **<0.001** | **a** | **b** |  |
|  | **B2** | **16.12** | **2** | **<0.001** | **a** | **a** | **b** |
|  | G1 | 4.5 | 2 | 0.105 | a | a | a |
|  | H1 | 0.54 | 2 | 0.762 | a | a | a |
|  | **H3** | **19.87** | **2** | **<0.001** | **a** | **b** | **a** |
| Fruits per plant | B1 | 1.83 | 1 | 0.176 | a | a |  |
|  | G1 | 2.06 | 2 | 0.357 | a | a | a |
|  | **H1** | **7.47** | **2** | **0.024** | **a** | **a** | **b** |
|  | H3 | 0.11 | 2 | 0.948 | a | a | a |
| Seeds per fruit | B1 | 0.18 | 1 | 0.674 | a | a |  |
|  | **G1** | **11.68** | **2** | **0.003** | **a** | **a** | **b** |
|  | H1 | 1.1 | 2 | 0.578 | a | a | a |
|  | H3 | 0.07 | 2 | 0.964 | a | a | a |
| Germination | B1 | 1.66 | 1 | 0.1971 | a | a |  |
|  | G1 | 0.35 | 2 | 0.8398 | a | a | a |
|  | H1 | 3.57 | 2 | 0.1681 | a | a | a |
|  | H3 | 3.88 | 2 | 0.1439 | a | a | a |
