# Supplementary material for: Flower color variation in Digitalis purpurea: Pollination and soil influences across native and introduced populations
Source: Am J Bot. 2026 Apr 3;113(4):e70186. doi: 10.1002/ajb2.70186 (PMC13103626; doi:10.1002/ajb2.70186)

**Appendix S10.** Spearman correlations between soil, plant size, and flower traits. (A) Correlogram showing significant positive or negative correlations. (B) Correlations between soil characteristics and plant and flower traits. *R* = Spearman rank correlation coefficient.


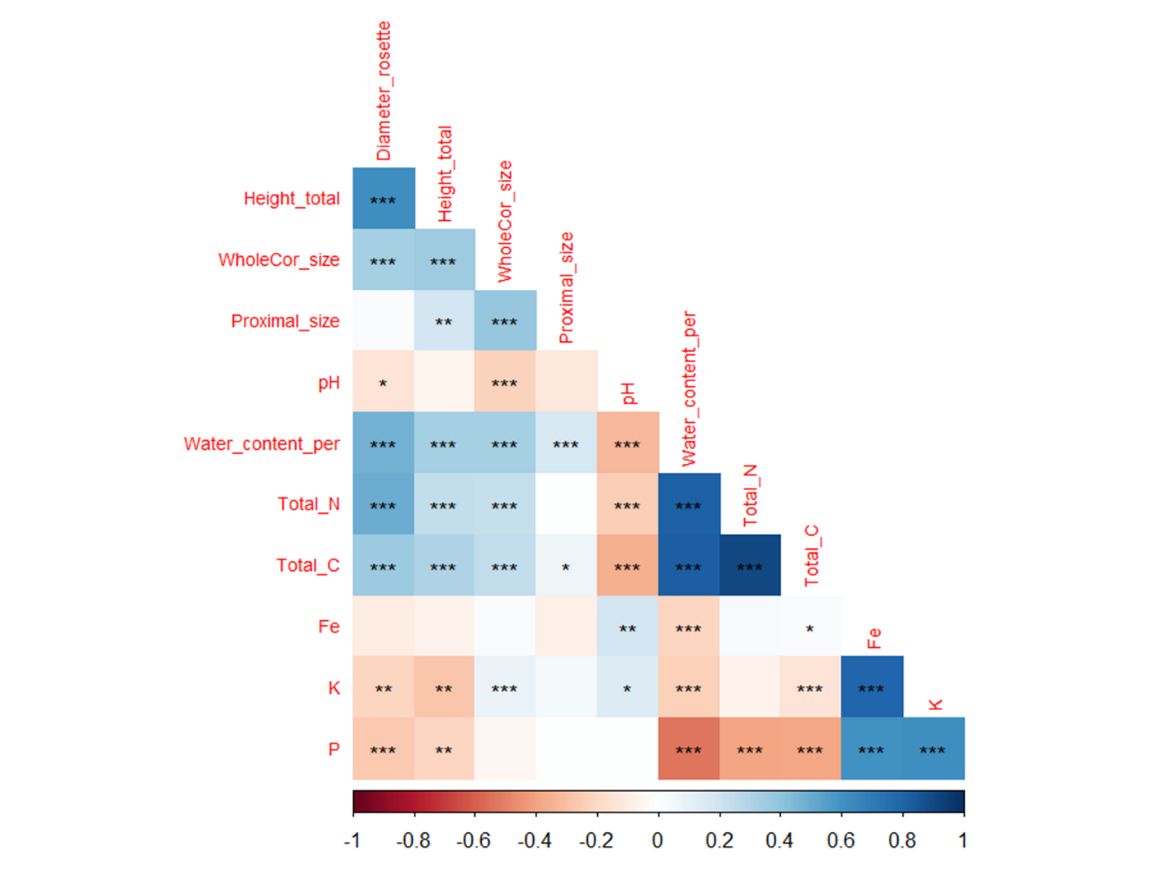


B

A


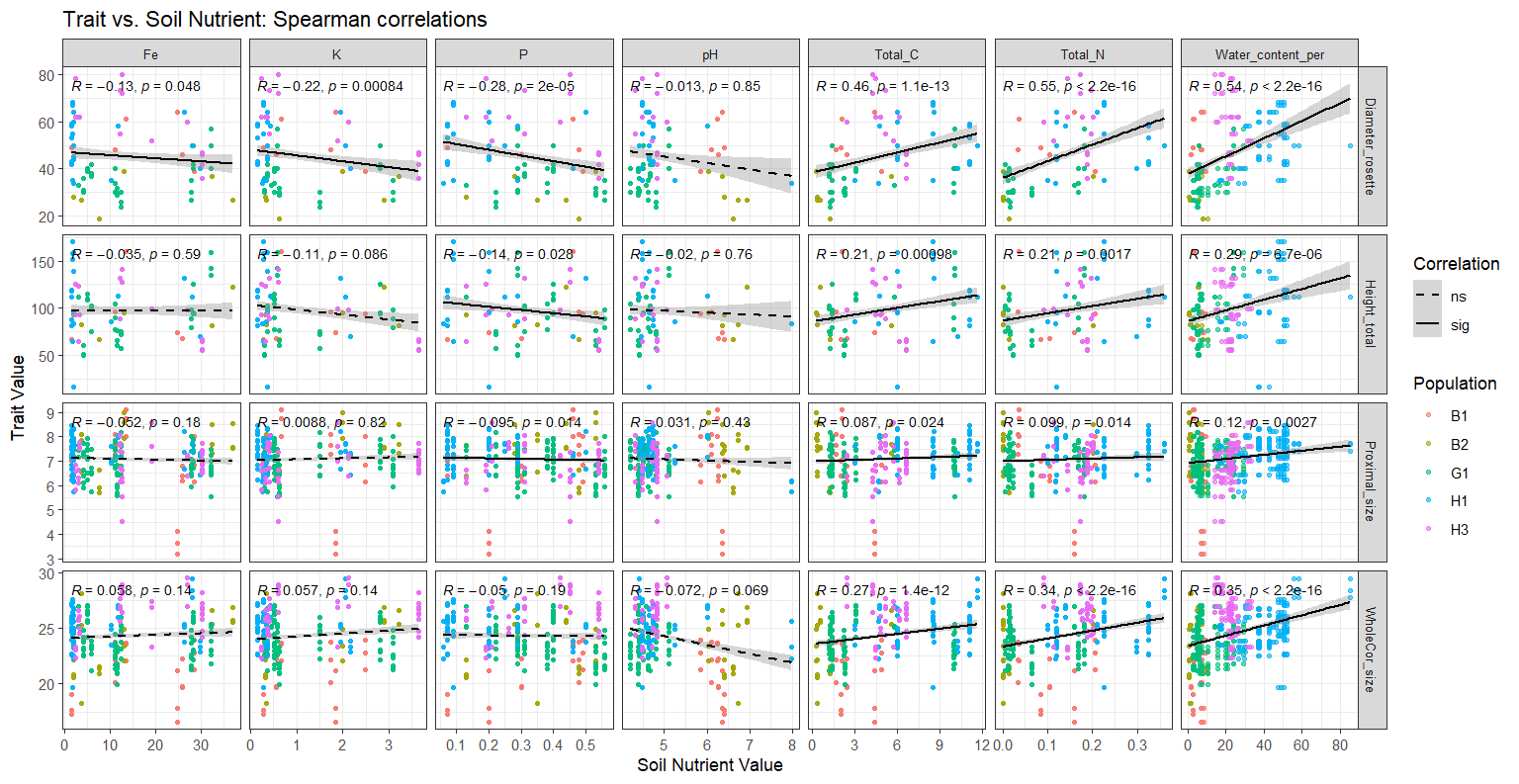

Supplement: Supplementary file 10 — Appendix S10. Spearman correlations between soil and plant and flower traits. [file AJB2-113-e70186-s005.docx]
