# Supplementary material for: Flower color variation in Digitalis purpurea: Pollination and soil influences across native and introduced populations
Source: Am J Bot. 2026 Apr 3;113(4):e70186. doi: 10.1002/ajb2.70186 (PMC13103626; doi:10.1002/ajb2.70186)

**Appendix S11.** Germination rate by flower color (violet, pink, and white) and population (introduced Bolivian, native Swedish) of *Digitalis purpurea*.


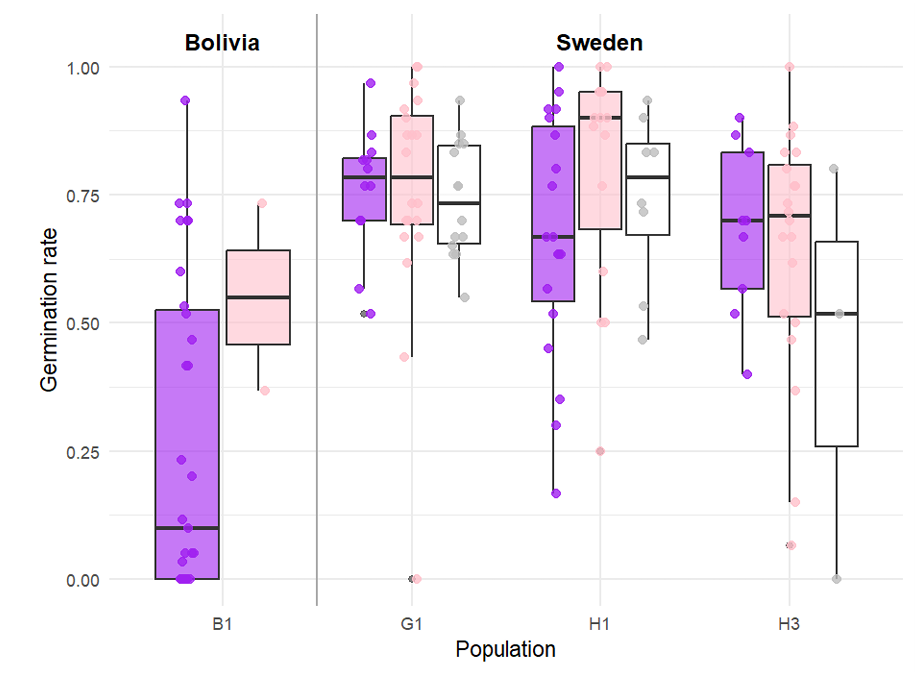

Supplement: Supplementary file 11 — Appendix S11. Germination rate per flower color and population. [file AJB2-113-e70186-s003.docx]
