# Supplementary material for: Flower color variation in Digitalis purpurea: Pollination and soil influences across native and introduced populations
Source: Am J Bot. 2026 Apr 3;113(4):e70186. doi: 10.1002/ajb2.70186 (PMC13103626; doi:10.1002/ajb2.70186)

**Appendix S12.** Soil pH and water content by population (Bolivian B1, B2; Swedish G1, H1, H3) and flower color morph (violet, pink, white) of *Digitalis purpurea*. (A) Soil pH varied between countries (~6 in Bolivia, 4–5 in Sweden. (B) Water content varied by population with low values in Bolivia and one population of Sweden. There was no significant difference by flower color in either pH or water content.


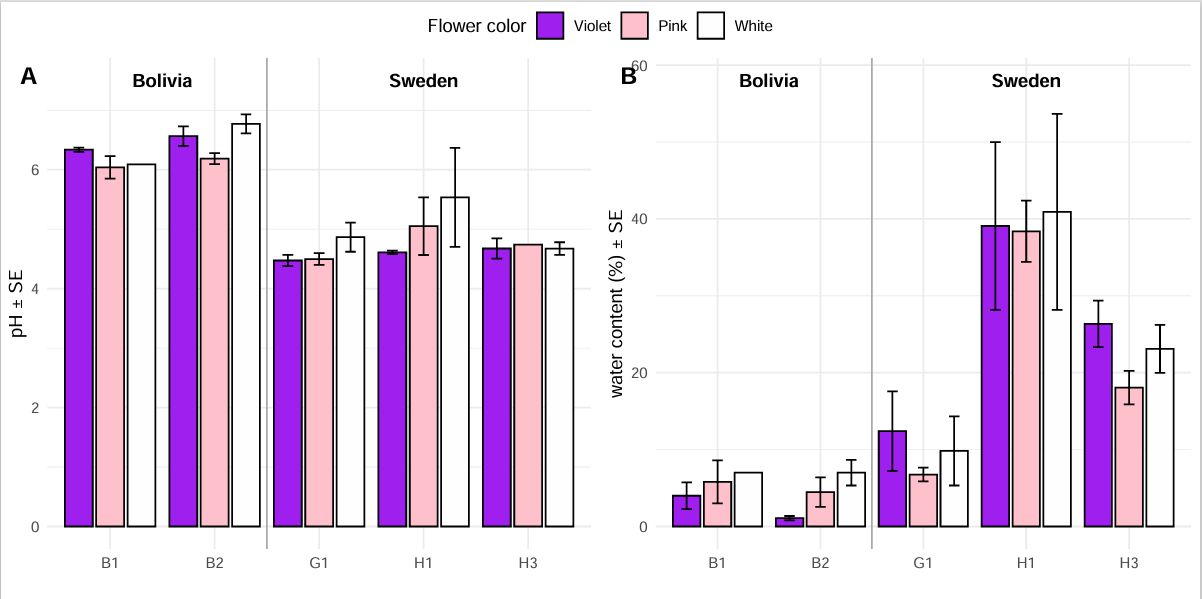

Supplement: Supplementary file 12 — Appendix S12. Soil pH and water content per population and flower color morph. [file AJB2-113-e70186-s012.docx]
